# FAK Mediated Coordination of Extracellular Matrix Remodeling and Cellular Mechanics Impairs Nanomedicine Delivery in Solid Tumors

**DOI:** 10.1002/advs.78146

**Published:** 2026-10-14

**Authors:** Tian Zhao, Hao Wu, Yujie Qin, Jingfei Zhu, Ye Huang, Wei Chen, Jiaxuan Yu, Xiang Qin, Shun Li, Junjie Li, Tingting Li, Yiyao Liu

**Affiliations:** ^1^ Department of Breast Surgery Sichuan Clinical Research Center for Cancer Sichuan Cancer Hospital & Institute Sichuan Cancer Center and School of Life Science and Technology University of Electronic Science and Technology of China Chengdu Sichuan People's Republic of China; ^2^ TCM Prevention and Treatment of Metabolic and Chronic Diseases Key Laboratory of Sichuan Province Hospital of Chengdu University of Traditional Chinese Medicine Chengdu Sichuan People's Republic of China; ^3^ Department of Otorhinolaryngology Hospital of Chengdu University of Traditional Chinese Medicine Chengdu Sichuan People's Republic of China; ^4^ Department of Urology Deyang People's Hospital Deyang Sichuan People's Republic of China; ^5^ The Department of Head and Neck Surgery Cancer Hospital of Shantou University Medical College Shantou Guangdong People's Republic of China

**Keywords:** clathrin‐mediated endocytosis, FAK, nanoparticle delivery, tumor extracellular matrix (ECM), tumor stroma remodeling

## Abstract

Tumor extracellular matrix (ECM) remodeling and cell‐intrinsic mechanical properties critically limit nanoparticle (NP) delivery, yet how these multiscale biophysical cues are coordinately regulated remains unclear. Here, we identify focal adhesion kinase (FAK/*PTK2*) as a central mechanobiological regulator that couples ECM remodeling with cellular mechanics to control NP transport and cellular uptake. The ECM derived from patients' tumors exhibited increased stiffness and collagen density, which severely restricted NP penetration, while *PTK2* emerged as a key integrin‐associated signaling hub linked to ECM organization. FAK depletion or inhibition disrupted ECM homeostasis, significantly enhanced NP penetration and cellular uptake. Mechanistically, FAK loss reduced cellular stiffness and cytoskeletal tension while increasing membrane deformability and transcriptionally reprogramming endocytic pathways, with clathrin‐mediated endocytosis as the dominant route. In vivo, FAK ablation remodeled the tumor stroma to enhance NP accumulation and penetration, and the combination of FAK inhibition with chemotherapy demonstrated potent antitumor efficacy. Together, these results establish a multiscale mechanistic paradigm in which FAK integrates ECM biophysics, cellular mechanics, and endocytic regulation to gatekeep nanomedicine delivery. Targeting FAK offers a potential strategy to enhance therapeutic penetration and efficacy in solid tumors.

## Graphical abstract

FAK orchestrates a mechanical barrier limiting nanomedicine accumulation in solid tumors by coupling ECM remodeling with cytoskeletal tension. Genetic or pharmacological FAK inhibition dismantles this barrier, enhances clathrin‐mediated uptake and penetration, and overcomes delivery limitations to improve antitumor efficacy.

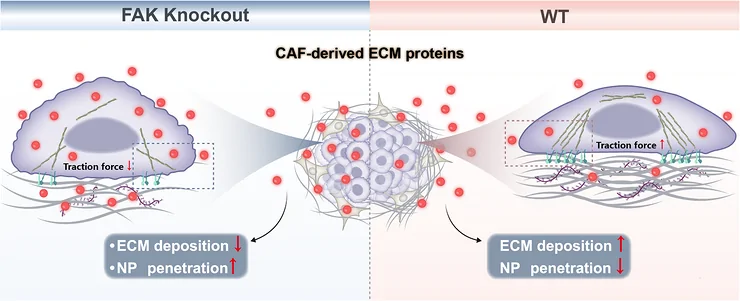

**Abbreviations**
AFM: Atomic force microscopy
AKT: Protein kinase B (also known as PKB)
CAF: Cancer‑associated fibroblast
CME: Clathrin‑mediated endocytosis
Cryo‑section: cryostat section (frozen section)
CAV1: Caveolin‐1
CLTC: Clathrin heavy chain
COL1A1: Collagen Type I
Cy5: Cyanine 5
DAB2: Disabled homolog 2
DOX: Doxorubicin
DEGs: Differentially expressed genes
DPBS: (Dulbecco's) phosphate‑buffered saline
ECM: Extracellular matrix
EGFR: Epidermal growth factor receptor
FAK: Focal adhesion kinase (gene name *PTK2*)
FN: Fibronectin
FBS: Fetal bovine serum
FTTC: Fourier transform traction cytometry
GO: Gene ontology
HA: Hyaluronic acid
IHC: Immunohistochemistry
ITGA/ITG: Integrin α/β subunit notation
LOX / LOXL: Lysyl oxidase / lysyl oxidase‑like
MFI: Mean fluorescence intensity
OCT: Optimal cutting temperature compound (embedding medium)
PAAm: Polyacrylamide (gel)
PEGPH20: PEGylated recombinant human hyaluronidase
PFA: Paraformaldehyde
PI3K: Phosphoinositide 3‑kinase
PIV: Particle image velocimetry
PLGA: Poly (lactic‑co‑glycolic acid)
PP: Polyethylenimine‐Poly (ethylene glycol)
P/S: Penicillin/streptomycin
PPI: Protein‐protein interaction
PS NPs: polystyrene nanoparticles
PYCARD: PYD and CARD domain containing
qPCR / RT‑qPCR: quantitative PCR/reverse‑transcription quantitative PCR
RNA‐seq: RNA sequencing
SDS: Sodium dodecyl sulfate
SEM: Scanning electron microscopy
siRNA: Small interfering RNA
TCGA: The cancer genome atlas
TME: Tumor microenvironment
TPM2: Tropomyosin 2
TFM: Traction force microscopy
Tunel: Terminal deoxynucleotidyl transferase dUTP nick‑end labeling
WT: Wild type

## 1 Introduction

The therapeutic potential of nanomedicines in oncology is fundamentally limited by a series of biological delivery barriers, among which the aberrant ECM of solid tumors constitutes a primary physical obstacle [1, 2]. During malignant progression, the stromal ECM undergoes pathological remodeling, becoming substantially stiffer and denser than healthy tissue [3, 4]. This increased rigidity results from the excessive deposition and dysregulated cross‐linking of structural proteins, primarily collagen and fibronectin, frequently catalyzed by enzymes like lysyl oxidases (LOXs) [3, 5, 6, 7, 8]. This biomechanical evolution generates a formidable, nanoscale mesh that severely restricts the convective and diffusive transport of therapeutic NPs [1]. For example, studies have shown that within dense collagen matrices mimicking desmoplastic tumors, NP penetration is often confined to just tens of micrometers, establishing steep peripheral drug gradients and leaving core regions undertreated [9, 10, 11]. Consequently, the pathological ECM is a major factor underlying the limited clinical success of many nanotherapies.

Beyond the interstitial matrix, cancer cells themselves present a secondary, cellular‐scale mechanical barrier. Cellular stiffness, dictated by the actomyosin cytoskeleton and focal adhesion complexes, directly influences the energetics of endocytic vesicle formation [12, 13, 14]. Stiffer cells, a hallmark of many carcinomas, typically exhibit elevated cortical tension, which can resist the membrane deformation necessary for efficient NP internalization via pathways such as clathrin‐mediated endocytosis [15, 16]. Conversely, cells with reduced stiffness or a dysregulated cytoskeleton may display altered endocytic activity [17, 18]. This establishes a dual‐challenge paradigm for nanodrug delivery: particles must first traverse the dense, stiff interstitial matrix before being efficiently internalized by frequently mechanically resistant cancer cells. A holistic strategy to improve nanotherapy must address both the extracellular transport barrier and the cellular uptake bottleneck.

Emerging evidence further indicates that these two mechanical barriers are not merely passive physical constraints but are actively regulated by cellular mechanotransduction pathways that directly shape cell‐nanoparticle interactions. Early studies have demonstrated that YAP, a central effector of the Hippo mechanotransduction pathway, regulates NP internalization and therapeutic efficacy in breast cancer cells by coordinating cell mechanics, membrane organization, and endocytic pathways [19]. More broadly, recent work has established mechanobiology as an important determinant of cell‐nanoparticle interactions, demonstrating that cellular mechanical state and mechanotransduction can influence nanoparticle binding, internalization, and intracellular trafficking [20]. These findings shift the conceptual framework from viewing mechanical properties simply as barriers to recognizing mechanotransduction signaling as an active regulatory layer that couples the physical state of the tumor cell to NP fate. However, the molecular mechanisms that connect mechanosensing pathways to both the extracellular matrix environment and the intracellular machinery responsible for NP uptake remain incompletely defined.

In breast cancer, the extracellular matrix (ECM) undergoes progressive stiffening, characterized by increased matrix rigidity and excessive deposition and remodeling of extracellular matrix components. The critical role of the ECM in limiting drug access has motivated the exploration of diverse TME‐normalization strategies, which are broadly categorized as enzymatic, pharmacological, or physical. One such enzymatic approach involves PEGPH20, a PEGylated recombinant human hyaluronidase, that degrades hyaluronan (HA) to reduce tumor interstitial pressure and enhance drug delivery [21]. Similarly, a first‐in‐class oral pan‐LOX inhibitor (PXS‐5505) has been shown to reduce desmoplasia and stiffness, improving perfusion and sensitizing tumors to chemotherapy [22, 23]. While these strategies have shown potential in modulating the ECM barrier, they often lack specificity and may induce adverse effects, including an increased metastatic risk. Therefore, a more targeted and promising approach would involve identifying and therapeutically modulating a central signaling hub that intrinsically governs both the synthesis and architecture of the tumor ECM and the mechanical phenotype of the cancer cells. Such a target could enable the coordinated “softening” of the dual extracellular and intracellular barriers.

FAK, encoded by the *PTK2* gene, emerges as a prime candidate for this integrative role. As a non‐receptor tyrosine kinase and a key component of focal adhesions, FAK resides at the interface between the ECM and the intracellular cytoskeleton, where focal adhesions serve as specialized structures that physically couple ECM proteins to the actin cytoskeleton. Through integrin‐mediated adhesion and FAK‐dependent signaling, these structures convert extracellular biochemical and mechanical cues into intracellular signaling and cytoskeletal responses. Thus, FAK functions as a critical regulator of mechanotransduction at the integrin‐ECM interface, coordinating ECM sensing, cytoskeletal organization, and cellular mechanical properties [24]. It is well‐established that FAK activation, triggered by matrices and integrin clustering, drives cancer progression, therapy resistance, and fibrosis by promoting ECM production and remodeling [25, 26]. Concurrently, FAK is a pivotal regulator of cytoskeletal dynamics and cellular tension through its control of Rho GTPase activity and actomyosin contractility [27]. Emerging evidence also suggests FAK signaling can modulate endocytic trafficking [28]. Thus, FAK resides at the intersection of the two principal delivery barriers: it is poised to regulate both the composition and stiffness of the interstitial ECM and the cytoskeletal stiffness and endocytic capacity of the cell. However, a systematic investigation into whether FAK functions as a central orchestrator that coordinately controls NP delivery by simultaneously regulating ECM barrier properties and cellular uptake mechanics remains to be undertaken. Elucidating this integrative mechanism is essential for developing a precise, mechanistically informed combinatorial strategy to enhance nanotherapeutic efficacy.

Our study aims to address how the breast tumor microenvironment is centrally regulated to impede nanotherapeutic delivery. We propose and investigate the hypothesis that FAK serves as a master regulator, coordinately controlling NP delivery by modulating extracellular matrix architecture, cellular mechanical properties, and endocytic activity. Through an integrated, multi‐scale experimental approach, we establish that FAK governs nanomedicine delivery through three synergistic axes: (1) transcriptionally reprogramming ECM composition to reduce stromal density, (2) dysregulating cytoskeletal organization to lower cellular stiffness, and (3) altering endocytic gene expression to promote uptake. These findings designate FAK as a master regulator of tumor permeability and provide a therapeutic target for stroma‐targeted delivery optimization.

## 2 Results

### 2.1 Tumor ECM Mechanical Properties Impede Nanoparticle Penetration

Breast tumors are characterized by a significantly stiffer ECM than normal mammary tissue [29, 30]. This biophysical feature is widely recognized to undermine the therapeutic efficacy of nanotherapeutic agents [1]. To systematically investigate the influence of the mechanical properties of tumor and normal mammary tissues on NP penetration behavior, we first performed mechanical characterization of patient‐derived tissues. The results revealed that tumor tissues exhibited markedly higher stiffness than normal mammary tissues (Figure 1A). These stiffness values are consistent with the pathological remodeling of tumor ECM and provide a validated experimental model for assessing NP penetration. To further elucidate the structural basis of breast tumors, we performed Masson's trichrome staining to visualize and quantify collagen. Staining results revealed a significantly higher collagen density in patient‐derived tumor ECM than in normal mammary ECM (Figure 1B,C). Additionally, quantitative analysis of collagen fiber orientation demonstrated a narrower distribution of orientation angles in tumor ECM, indicating a high degree of collagen fiber alignment (Figure 1D), suggesting matrix architectural remodeling associated with tumor progression [31, 32].

**FIGURE 1:**
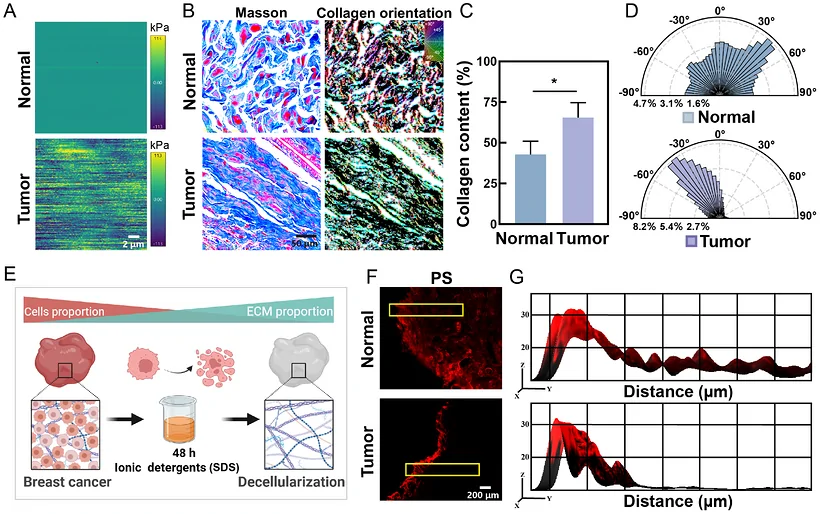
Tumor ECM mechanical heterogeneity restricts NP penetration in the tumor tissues of breast cancer patients. (A) AFM‐based nanomechanical maps (elastic modulus) of tumor and normal tissue. (B) Masson's trichrome staining of tumor and normal tissues, highlighting collagen fibers in blue. Scale bar, 50 µm. (C) Quantitative analysis of total collagen content. Data are shown as the mean ± SD; ^*^ *p* < 0.05. (D) Circular rose plots showing collagen fiber orientation. (E) Schematic illustrating the decellularization process to isolate pure ECM scaffolds. (F) Fluorescence images showing the penetration of red fluorescent NPs into tumor and normal ECM scaffolds. The yellow boxes indicate regions of interest (ROIs). Scale bar, 200 µm. (G) Fluorescence intensity profiles corresponding to the ROIs in (F), plotting signal versus penetration depth.

To systematically investigate the influence of the mechanical properties of tumor‐derived ECM versus normal mammary ECM on NP penetration behavior, we first subjected fresh breast tumors and matched adjacent normal tissues to a decellularization protocol to isolate the ECM scaffolds (Figure 1E). We employed Cyanine 5 (Cy5)‐labeled polystyrene (PS) NPs as a model nanotherapeutic system to evaluate NP distribution within the decellularized ECM scaffolds. Throughout the normal mammary ECM scaffold, we observed strong PS NP fluorescence intensity, along with deep penetration and a gradual decay in fluorescence signal from the tissue surface to the interior (Figure 1F). This observation is attributed to the porous, interconnected structural architecture of normal mammary ECM, which creates unobstructed channels to facilitate NP diffusion. By comparison, the overall PS NP fluorescence of ECM derived from tumors was found to be weak, and a steep exponential decay in fluorescence signal was observed along the penetration axis (Figure 1G). These results collectively confirm that the dense, compact structural nature of tumor ECM acts as a physical barrier that impedes NP translocation. In summary, the biophysical remodeling of breast tumor ECM is characterized by the synergistic effects of increased stiffness, elevated collagen density, and an organized, aligned structural architecture. This collectively acts to reduce the delivery efficiency of NPs. These findings imply that ECM mechanical remodeling is driven by specific upstream signaling axes. We therefore interrogated the underlying molecular regulatory network.

### 2.2 PTK2 Orchestrates Extracellular Matrix Remodeling to Drive Breast Cancer Progression

To investigate the functional role of *PTK2* in the breast tumor microenvironment and its potential linkage to extracellular matrix dynamics, we conducted an integrated, multi‐pronged study employing protein‐protein interaction (PPI) network mapping, GO enrichment analysis, transcriptomic profiling, and immunohistochemical validation. PPI network analysis identified *PTK2* as a central hub within a multilayered interaction network, exhibiting extensive connectivity with key signaling molecules, including SRC and EGFR (Figure 2A). GO enrichment analysis further revealed that *PTK2*‐associated genes are predominantly enriched in integrin‐mediated signaling, cell adhesion, and cell‐matrix adhesion pathways, with integrin signaling displaying the highest enrichment intensity, supporting a role for *PTK2* in ECM‐related signal transduction (Figure 2B).

**FIGURE 2:**
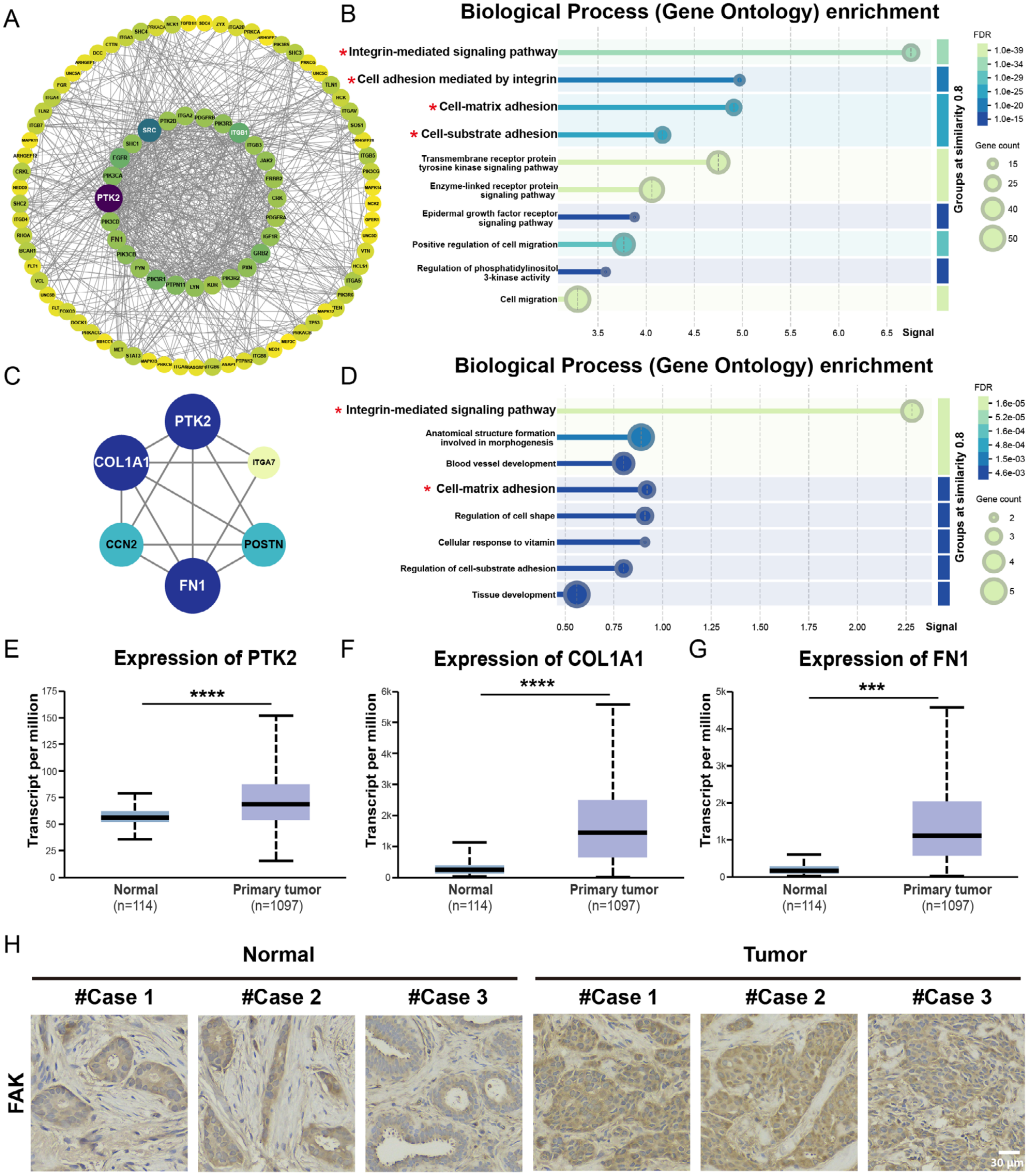
Analysis of *PTK2* (FAK) interaction networks, pathway enrichment, and expression in breast cancer. (A) PPI network of *PTK2* (FAK). The PPI network was constructed using the STRING database. (B) GO biological process enrichment analysis of the associated genes in (A) was performed to identify significantly enriched biological pathways. (C) Interaction network focusing on *PTK2* (FAK) with ECM‐related proteins; the network highlights functional associations between *PTK2* and key ECM components/integrin subunits. (D) Corresponding GO enrichment analysis of (C). (E) Analysis of *PTK2* gene expression based on RNA‐sequencing data from the TCGA breast invasive carcinoma (BRCA) cohort (normal *n* = 114, primary tumor *n* = 1097). An unpaired two‐sample *t*‐test was employed. *PTK2* was significantly upregulated in primary tumors (^****^ *p* < 0.0001). (F) Analysis of COL1A1 gene expression based on RNA‐sequencing data from TCGA BRCA cohort (normal *n* = 114, primary tumor *n* = 1097). An unpaired two‐sample *t*‐test was employed. COL1A1 was significantly upregulated in primary tumors (^****^ *p* < 0.0001). (G) Analysis of FN1 gene expression based on RNA‐sequencing data from the TCGA BRCA cohort (normal *n* = 114, primary tumor n = 1097). An unpaired two‐sample *t*‐test was employed. FN1 was significantly upregulated in primary tumors (^***^ *p* < 0.001). Gene expression levels were compared between primary tumor samples and solid tissue normal samples. (H) Representative immunohistochemistry (IHC) staining of *PTK2* (FAK) in human normal breast tissue and breast cancer tissue. Scale bar, 30 µm.

Focused interrogation of ECM‐associated interactions demonstrated that *PTK2* forms a tightly interconnected module with major matrix components, including COL1A1, FN1, POSTN, and CCN2, as well as the integrin subunit ITGA7 (Figure 2C). This module is significantly enriched in biological processes related to cell‐matrix adhesion and tissue morphogenesis (Figure 2D). Comparative transcriptomic data from The Cancer Genome Atlas (TCGA) breast cancer cohort revealed that *PTK2* expression levels in primary tumors were mildly elevated compared to normal tissues (Figure 2E). More notably, the expression of downstream matrix genes COL1A1 and FN1 was significantly upregulated in tumors (Figure 2F,G). This differential expression pattern suggests that *PTK2* may synergistically activate matrix synthesis programs through transcriptional regulation or signal amplification mechanisms, thereby driving ECM remodeling in breast cancer. Consistently, immunohistochemical analysis confirmed elevated FAK protein (*PTK2*) expression in tumor tissues (Figure 2H). Collectively, these findings identify *PTK2* as a central signaling hub in ECM‐associated pathways and support its relevance as an indicator of adverse prognosis in breast cancer. Given the hub position of *PTK2* within ECM‐associated networks, we next validated its functional relevance and mechanistic dependence at the cellular level.

### 2.3 FAK Regulates Extracellular Matrix Homeostasis via AKT Signaling

FAK is a key regulator of ECM dynamics and mechanotransduction. In this study, we aimed to examine the role of FAK in regulating ECM composition in MCF‐7 cells and tumor spheroids using a multi‐method approach. We hypothesized that FAK depletion would significantly alter ECM protein deposition, potentially influencing tumor aggressiveness and cell adhesion.

To define the role of *PTK2*‐encoded FAK in regulating ECM protein expression and associated signaling pathways in breast cancer cells, we performed immunofluorescence analysis of FN1 and COL1A1 in MCF‐7 cells. FAK depletion markedly reduced COL1A1 signal intensity (Figure 3A; Figure S1A), consistent with the corresponding quantitative analysis (Figure S1B); whereas the strength of the FN signal did not change significantly (Figure 3B; Figure S1C), consistent with the corresponding quantitative analysis (Figure S1D). These data indicate differential regulation of ECM components following FAK depletion. Gene expression analysis further demonstrated upregulation of FN1 alongside downregulation of COL1A1 and other ECM‐associated genes (Figure 3C). Western blot confirmed the complete loss of FAK protein and showed a marked reduction in pPI3K and pAKT protein levels, consistent with suppression of PI3K/AKT signaling activity (Figure 3D).

**FIGURE 3:**
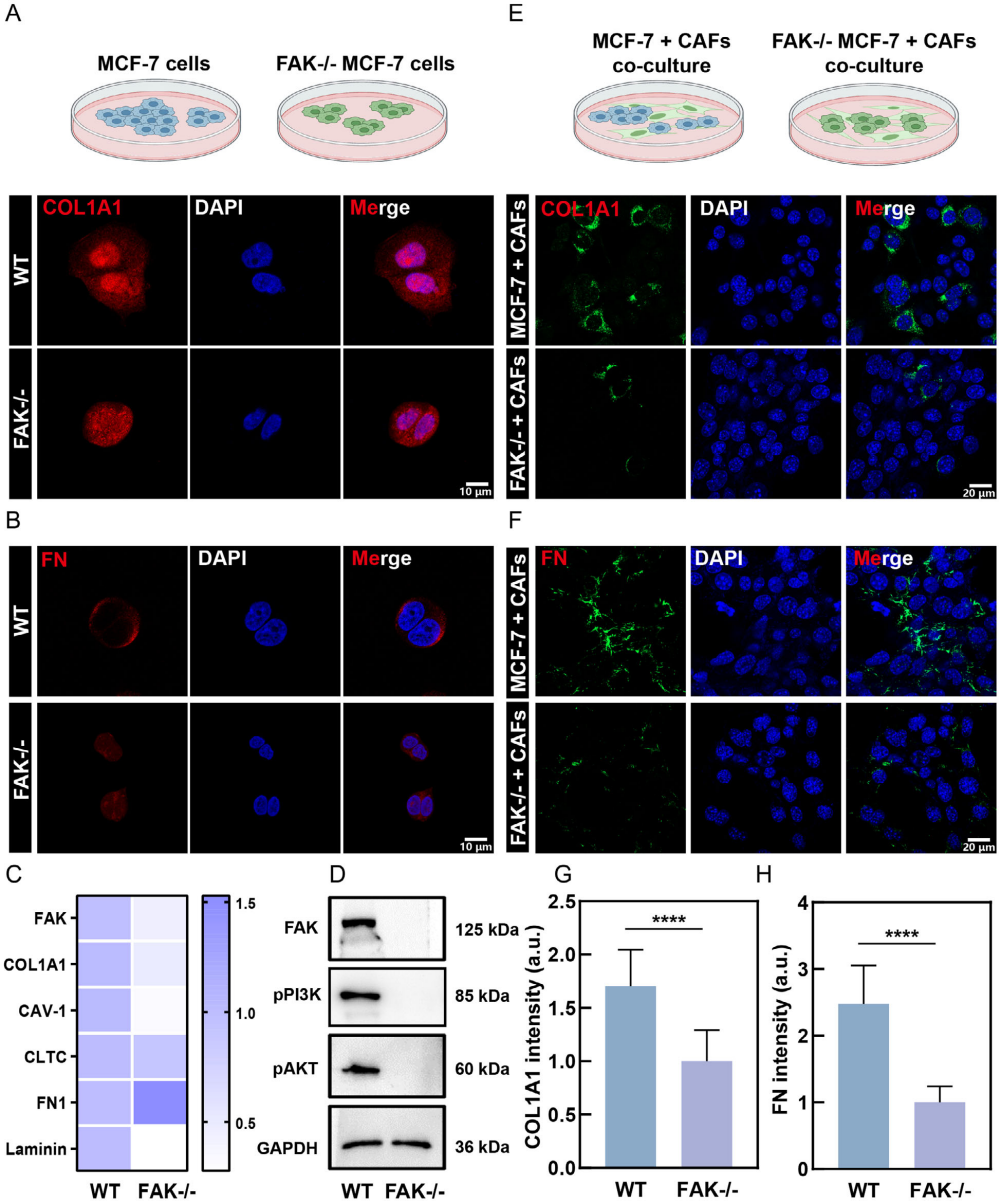
FAK modulates ECM protein expression, downstream signaling, and the tumor microenvironment. (A) Schematic illustration of WT or FAK‐/‐ MCF‐7 cells monoculture. Immunofluorescence staining of COL1A1 (red) in WT and FAK‐/‐ MCF‐7 cells, counterstained with DAPI (blue). Scale bar, 10 µm. (B) Immunofluorescence staining of FN (red) in WT and FAK‐/‐ MCF‐7 cells, counterstained with DAPI (blue). Scale bar, 10 µm. (C) Heatmap depicting relative mRNA expression levels of ECM‐related and endocytosis‐associated genes in WT versus FAK‐/‐ MCF‐7 cells, as determined by *q*PCR analysis (*n* = 3 independent experiments). (D) Western blot analysis of FAK, pPI3K, and pAKT protein levels in WT and FAK‐/‐ MCF‐7 cells. GAPDH served as a loading control. (E) Schematic of co‐culture systems consisting of MCF‐7 cells and ‌cancer‐associated fibroblasts‌ (CAFs), with or without FAK expression. Representative immunofluorescence images showing COL1A1 (green) expression in WT or FAK‐/‐ MCF‐7 cells co‐cultured with CAFs. Nuclei are stained with DAPI (blue). Scale bar, 20 µm. (F) Representative immunofluorescence images showing FN (green) expression in WT or FAK‐/‐ MCF‐7 cells co‐cultured with CAFs. Nuclei are stained with DAPI (blue). Scale bar, 20 µm. (G) Quantification of immunofluorescence intensity for COL1A1 from images in (E). (H) Quantification of immunofluorescence intensity for FN from images in (F). Data are presented as mean ± SD (*n* = 6 fields of view per group from two independent experiments); ^****^ *p* < 0.0001, unpaired *t*‐test.

To evaluate the role of FAK in a multicellular microenvironment, we employed a co‐culture model of the MCF‐7 breast cancer cell line and primary cancer‐associated fibroblasts (CAFs) isolated from breast cancer patients at the Affiliated Cancer Hospital of UESTC. Immunofluorescence analysis showed that both FN1 and COL1A1 signals were substantially reduced following FAK knockout in MCF‐7 cells, accompanied by disruption of FN1 fibrillar networks and decreased extracellular deposition of COL1A1 (Figure 3E,F; Figure S2). Quantitative analysis confirmed significant reductions in fluorescence intensity for both ECM proteins (Figure 3G,H). These results indicate that FAK activity is positively associated with ECM protein abundance in a complex cellular environment.

In summary, FAK plays a pivotal role in ECM regulation in MCF‐7 cells and multicellular co‐culture models. Its depletion alters ECM composition, reducing COL1A1 in monoculture and both COL1A1 and FN in the MCF‐7/CAF co‐culture model. This work underscores the importance of FAK in regulating ECM homeostasis, with implications for understanding its role in tumor progression and metastasis. Although FAK‐mediated ECM regulation is established at the cellular level, its integrated impact on multiscale nanoparticle transport within 3D architectures remains to be defined.

### 2.4 Extracellular Matrix Architecture and FAK Signaling Cooperatively Control Nanoparticle Penetration and Uptake in Breast Cancer Models

To define how ECM architecture and FAK signaling regulate nanoparticle transport, we established different three‐dimensional tumor spheroid models. MCF‐7 was used to define the tumor‐cell‐intrinsic molecular and mechanobiological mechanisms of FAK‐mediated ECM regulation, whereas 4T1 was used to evaluate the consequences of this mechanism in a more aggressive 3D and immunocompetent tumor setting, particularly in the context of NP delivery and therapeutic efficacy. We first fabricated fibronectin (FN)‐coated micropatterned substrates (Figure 4A). We then generated 4T1/NIH3T3 mixed tumor spheroids on these patterns and treated them with the FAK inhibitor PF‐573228 (2 µm). Immunofluorescence analysis revealed a significant reduction in COL1A1 protein expression within the inhibitor‐treated spheroids compared to the control group (Figure 4B). Quantitative analysis confirmed a significant reduction in mean fluorescence intensity after treatment with the FAK inhibitor (Figure 4C). In the embedded spheroid model, pre‐formed spheroids were embedded in collagen matrices of different densities (0.5 mg/mL and 2 mg/mL). Reflectance imaging showed that low‐density collagen formed a loose fibrillar network, while high‐density collagen formed a compact structure. Corresponding fluorescence imaging demonstrated widespread NP distribution in low‐density matrices, whereas NP penetration was restricted to the spheroid periphery under high‐density conditions (Figure S3). Consistent with the diffusion behavior observed in acellular collagen hydrogels (Figure S4), these results indicate that dense extracellular collagen matrices act as physical barriers that limit NP penetration into tumor spheroids.

**FIGURE 4:**
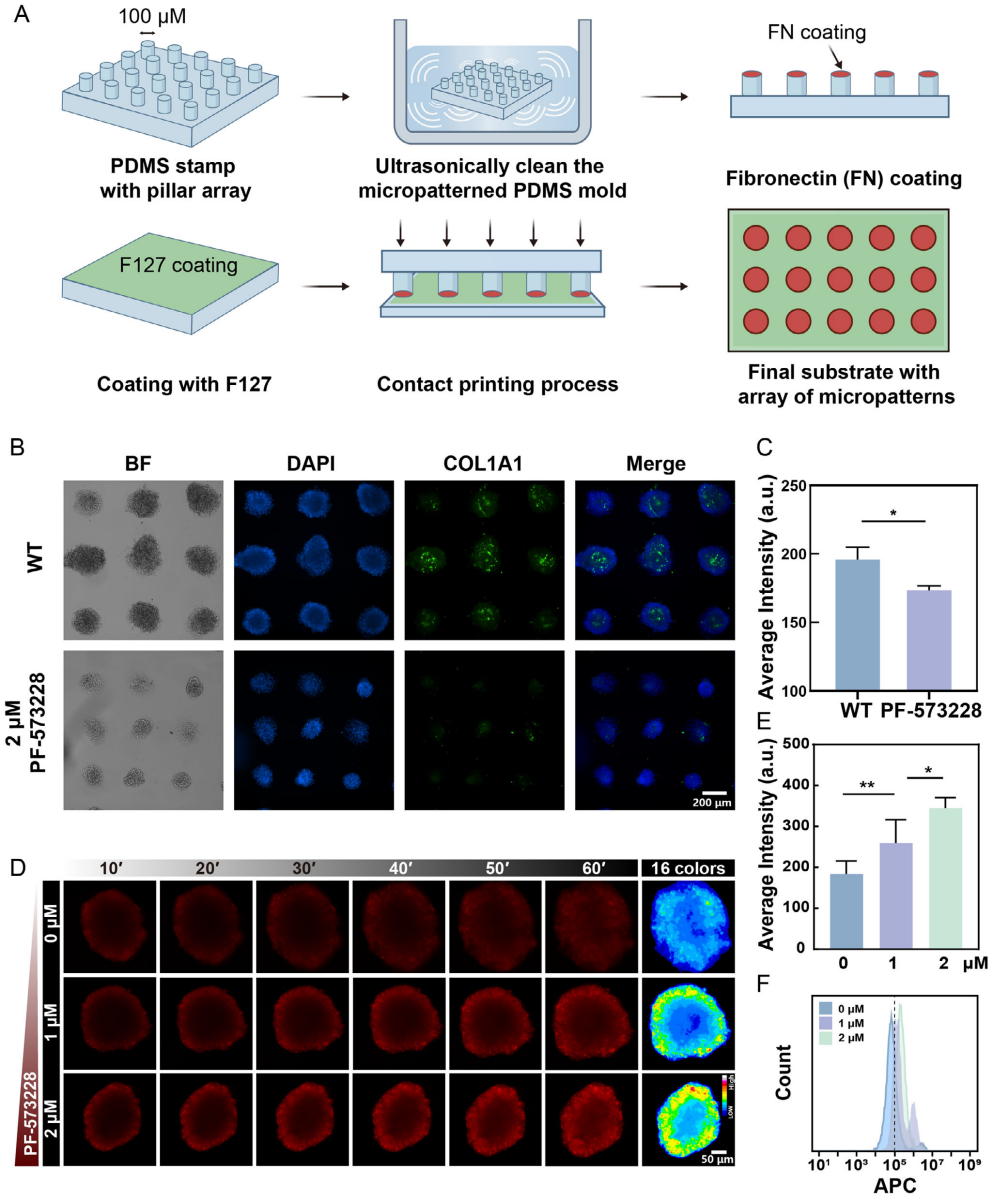
FAK inhibition on NP penetration and cellular uptake in tumor spheroids. (A) Schematic illustration of the fabrication process for generating FN‐coated micropatterned substrates. (B) Immunofluorescence characterization of COL1A1 expression in 4T1/NIH3T3 mixed tumor spheroids generated by micropatterning. Spheroids were treated with or without the FAK inhibitor PF‐573228 (2 µm). Representative images show COL1A1 (green), nuclei stained with DAPI (blue), merged channels, and corresponding bright‐field (BF) images. Scale bar, 200 µm. (C) Quantification of COL1A1 fluorescence intensity in tumor spheroids from (B). Data are presented as average fluorescence intensity (a.u.); mean ± SD. Statistical analysis was performed using an unpaired *t*‐test. ^*^ *p* < 0.05. (D) Representative fluorescence images of NP penetration in spheroids pretreated with the FAK inhibitor PF‐573228 at indicated concentrations (0, 1, 2 µm), followed by NP incubation. Penetration was monitored over 60 min. Corresponding pseudocolor images are shown. 0 µm denotes the untreated control, and the red channel shows the distribution of Cy5‐PS nanoparticles. Scale bar, 50 µm. (E) Quantification of average NP fluorescence intensity within inhibitor‐treated spheroids at 60 min. Data: mean ± SD (*n* = 3 spheroids/group). Statistical analysis was performed using one‐way ANOVA with appropriate post hoc tests. ^*^ *p* < 0.05, ^**^ *p* < 0.01. (F) Flow cytometry analysis of NP uptake in 4T1 tumor spheroids treated with increasing concentrations of PF‐573228 (0, 1, 2 µm). Histograms show the intracellular fluorescence distribution.

To specifically determine the tumor cell‐intrinsic contribution of FAK signaling to nanoparticle transport, we treated monocultured 4T1 cell spheroids with the FAK inhibitor PF‐573228. Fluorescence imaging showed a dose‐dependent increase in NP penetration and more homogeneous distribution (Figure 4D). Quantitative analysis confirmed increased fluorescence intensity with inhibitor concentration (Figure 4E). Flow cytometry further demonstrated that FAK inhibition significantly enhanced NP uptake (Figure 4F). These findings support a critical role for FAK signaling in restricting NP transport and cellular uptake within tumor spheroids. Collectively, these findings demonstrate that ECM densification and FAK activation cooperate to establish physical and cellular barriers that limit NP delivery within tumor spheroids. As tissue‐level transport and cellular uptake are governed by distinct physical constraints, we next examined the mechanobiological basis of FAK‐dependent endocytic regulation.

### 2.5 FAK Coordinates Multiscale Cellular Mechanics to Modulate Nanoparticle Endocytosis

To investigate whether FAK‐dependent mechanobiological properties regulate NP internalization, we systematically assessed cellular uptake alongside mechanical phenotypes using complementary imaging and biophysical approaches. Fluorescence microscopy revealed that MCF‐7 cells formed compact aggregates with weak and heterogeneous intracellular NP signals. In contrast, FAK knockout (FAK‐/‐) and PF‐573228‐treated cells exhibited a more dispersed morphology and increased, homogeneous intracellular fluorescence (Figure 5A). Quantitative analysis of the fluorescence images, performed by measuring the fluorescence intensity of cellular clusters within each image field, further demonstrated significantly higher cellular fluorescence in both FAK‐/‐ and PF‐573228‐treated cells compared with WT cells, whereas no significant difference was observed between the FAK‐/‐ and PF‐573228‐treated groups (Figure 5B). These results indicate that genetic knockout and pharmacological inhibition of FAK similarly enhance intracellular NP‐associated fluorescence. Flow cytometry further confirmed a significant increase in cellular fluorescence in FAK‐/‐ cells, as reflected by the rightward shift in the APC fluorescence distribution (Figure 5C), supporting enhanced NP uptake following FAK depletion. Although the magnitude of these increases was moderate, their reproducibility across independent experiments and consistency between FAK depletion and pharmacological inhibition support their biological relevance.

**FIGURE 5:**
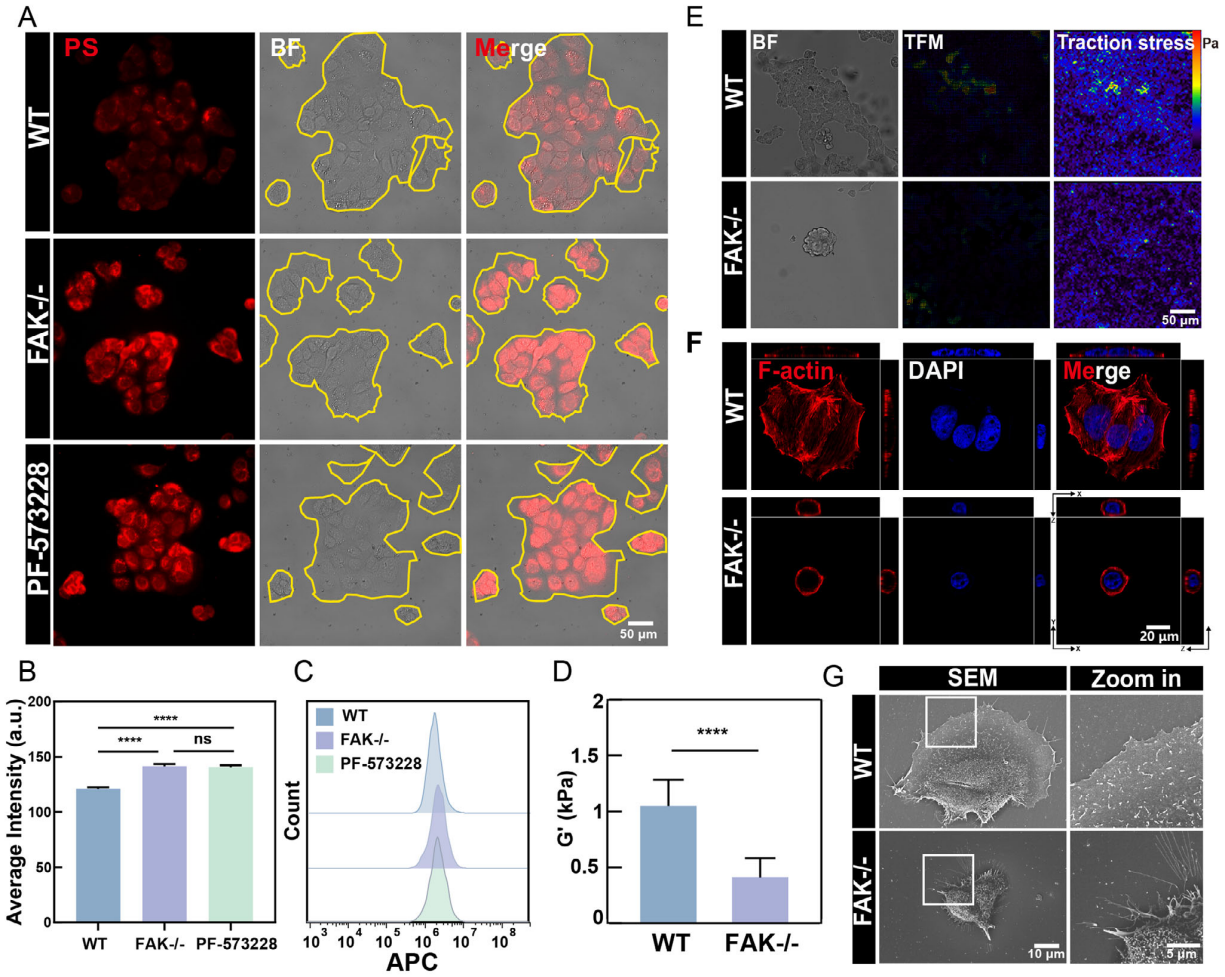
FAK regulates NP internalization and cellular phenotypes in breast cancer cells. (A) Fluorescence microscopy images of MCF‐7 cells, comparing WT and FAK‐knockout (FAK‐/‐) cell lines, and PF‐573228‐treated MCF‐7 cells. Cells were imaged in PS (red fluorescence), bright‐field (BF), and merged channels. Scale bar, 50 µm. (B) Quantification of fluorescence intensity in cellular clusters. Fluorescence intensity was measured for cellular clusters in 5 randomly selected fields per group. Data are presented as mean ± SD from 3 independent experiments (*n* = 15 fields). Statistical significance was calculated using one‐way ANOVA with Tukey's post hoc test. (C) Flow cytometry analysis of intracellular fluorescence intensity; histograms represent the distribution of APC channel fluorescence signals. (D) Quantification of cellular mechanical stiffness in MCF‐7 cells using nanoindentation. The storage modulus (G′) was measured for WT and FAK‐/‐ cells and presented as individual data points with mean ± SD. Statistical analysis was performed using an unpaired *t*‐test, ^****^ *p* < 0.0001. (E) Traction force microscopy (TFM) analysis of MCF‐7 cells cultured on compliant substrates. Representative bright‐field (BF), traction force maps (TFM), and corresponding traction stress heatmaps are shown for WT and FAK‐/‐ cells. Scale bar, 50 µm. (F) Confocal laser scanning microscopy three‐view diagrams of cytoskeletal organization in MCF‐7 cells. F‐actin was stained in red and nuclei were labeled with DAPI (blue) in WT and FAK‐/‐ cells. Scale bar, 20 µm. (G) SEM images showing the surface ultrastructure of WT and FAK‐/‐ MCF‐7 cells. Insets indicate magnified regions of the cell surface. Scale bars, 10 µm (left) and 5 µm (right).

To elucidate the underlying mechanisms, we quantified cellular stiffness using nanoindentation. WT cells exhibited a higher storage modulus (G'), whereas FAK‐deficient cells showed a pronounced reduction in stiffness (Figure 5D). Traction force microscopy demonstrated that WT cells generated strong and spatially uniform traction stresses, while FAK‐/‐ cells displayed reduced and heterogeneous force distributions (Figure 5E). Confocal imaging revealed well‐organized F‐actin stress fibers in WT cells, which became disrupted and diffuse upon FAK depletion (Figure 5F; Figure S5A). Quantitative analysis showed that cell area was significantly reduced upon FAK depletion (Figure S5B). Consistently, scanning electron microscopy (SEM) showed that WT cells maintained relatively smooth surfaces, whereas FAK‐/‐ cells exhibited abundant membrane surface microvilli and increased surface roughness (Figure 5G; Figure S5C). Microvilli are actin‐rich finger‐like protrusions that not only participate in mechanosensing and signal transduction but also regulate integrin clustering and adhesion maturation by increasing local curvature. Indeed, through the laminin/integrin β1/FAK/actin axis, FAK‐mediated mechanical signaling pathways directly respond to microenvironmental stiffness changes and guide cytoskeletal reorganization [33, 34]. Deletion of FAK leads to the disruption of mechanical signals, which subsequently causes stress fiber depolymerization. This relieves normal constraints on the membrane skeleton, allowing the tension‐regulated cortical actin network to relax. This mechanical release likely contributes to aberrant microvillus hyperplasia and enhanced membrane deformability, thereby creating a surface topology more permissive for NP binding and internalization.

Collectively, these results demonstrate that FAK regulates NP internalization by modulating cellular mechanical states. Loss of FAK reduces cellular stiffness, weakens traction forces, and disrupts cytoskeletal organization, accompanied by membrane remodeling, all of which are associated with enhanced NP uptake. Notably, this study shifts the mechanistic perspective from classical receptor‐mediated uptake toward a mechanics‐centered framework, in which global cellular physical properties constrain endocytic capacity. Although the specific endocytic pathways remain to be defined, these findings are consistent with emerging evidence that membrane deformability and cytoskeletal tension critically influence vesicle formation. Following the identification of mechanotransductive regulation, we next performed transcriptomic profiling to define the transcriptional network reprogramming induced by FAK loss.

### 2.6 FAK Serves as a Central Transcriptional Regulator That Coordinates Distinct Endocytic Pathways

To delineate the transcriptional regulatory role of FAK in breast cancer cells, we performed RNA sequencing (RNA‐seq) to compare FAK‐/‐ and WT MCF‐7 cells. Differential expression analysis identified a total of 3,851 differentially expressed genes (DEGs), including 2,504 upregulated and 1,347 downregulated genes in FAK‐/‐ cells (adjusted *p* < 0.05). The volcano plot illustrates the global distribution of DEGs, with upregulated genes shown in red and downregulated genes in blue, plotted against log2 fold change and −log10 adjusted *P* values. Several representative genes were highlighted. Genes related to cytoskeletal organization and contractile regulation, including MYO15B and TPM2, were upregulated, whereas genes associated with cell adhesion and endocytic signaling, such as DAB2, were downregulated (Figure 6A).

**FIGURE 6:**
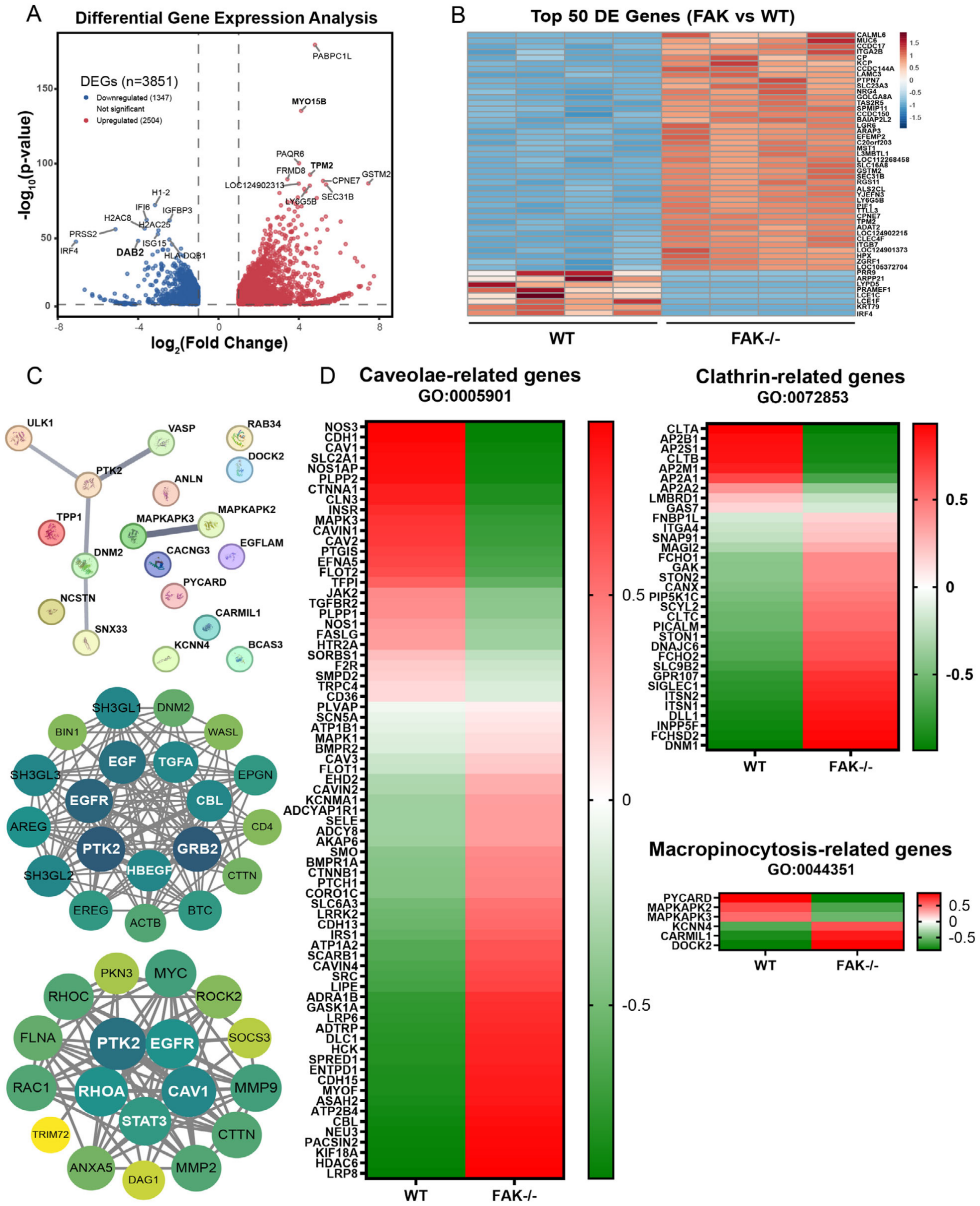
Transcriptomic profiling and network analysis of FAK‐regulated endocytic pathways. (A) Volcano plot of differentially expressed genes (DEGs) between FAK‐/‐ and WT MCF‐7 cells from RNA‐sequencing data. Genes with a |log2(fold change) | > 1 and adjusted *p*‐value < 0.05 are highlighted in color (red: upregulated; blue: downregulated). Selected key DEGs are annotated. (B) Heatmap representation of the top 50 differentially expressed genes between WT and FAK‐/‐ cells identified from RNA‐seq analysis. Gene expression values were normalized and clustered across samples to visualize transcriptional variation. (C) PPI network constructed using the STRING database based on differentially expressed genes. The network is centered on *PTK2* (FAK) and visualized to illustrate interactions among proteins associated with macropinocytosis, caveolae‐mediated endocytosis, and clathrin‐mediated endocytosis. (D) Heatmap analysis of gene expression profiles associated with major endocytic pathways, including macropinocytosis, caveolae‐mediated endocytosis, and clathrin‐mediated endocytosis, in WT and FAK‐/‐ cells. Gene sets were curated based on known pathway components and visualized following normalization. Rows represent genes grouped by pathway, and columns represent samples.

To further characterize transcriptomic alterations, we performed unsupervised hierarchical clustering of the top 50 DEGs ranked by a combined score of log2FC and statistical significance. The heatmap revealed clear segregation between FAK‐/‐ and WT samples, with widespread upregulation observed in FAK‐/‐ cells and corresponding lower expression in WT controls, indicating extensive transcriptional reprogramming upon FAK deletion (Figure 6B). These findings suggest that FAK loss broadly alters the transcriptional state of MCF‐7 cells.

To investigate the molecular interaction network associated with FAK, we constructed a PPI network using the STRING database. The network centered on *PTK2* (FAK) revealed multiple functional modules. The first module included ULK1, VASP, RAB34, and DOCK2, associated with autophagy and vesicle trafficking, along with MAPKAPK2, MAPKAPK3, and PYCARD, which are linked to macropinocytosis. The second module comprised key components of clathrin‐mediated endocytosis, including SH3GL3, DNM2, WASL, and CBL. The third module comprised proteins involved in cytoskeletal remodeling and membrane dynamics. It connected key components of caveolin‐mediated endocytosis, such as RHOA, RAC1, CAV1, and MMP9. RAC1 showed close connectivity with FAK (Figure 6C). These results indicate that FAK may function as a central node integrating multiple endocytic pathways.

To further assess the role of FAK in endocytosis, we analyzed gene expression changes across three major endocytic pathways: macropinocytosis, caveolin‐mediated endocytosis, and clathrin‐mediated endocytosis. In the clathrin‐mediated pathway (GO:0072853), CLTC and ITSN2 exhibited increased expression. In the caveolin‐mediated pathway (GO:0005901), genes including EHD2 and PACSIN2 showed elevated expression levels in FAK‐/‐ cells. In the macropinocytosis pathway (GO:0044351), genes such as PYCARD and MAPKAPK2 were upregulated in FAK‐/‐ cells (Figure 6D). These findings demonstrate that FAK deletion is associated with transcriptional alterations in genes related to multiple endocytic pathways. To functionally validate the contribution of the endocytic pathways implicated by the transcriptomic analysis, we next examined the effects of pharmacological inhibitors on fluorescent PS NP uptake in MCF‐7 cells by flow cytometry. In control cells, the NPs‐associated fluorescence distribution progressively shifted toward higher fluorescence intensities from 2 to 4 and 6 h, indicating time‐dependent intracellular accumulation of NPs. Treatment with Dynasore, a dynamin inhibitor, substantially attenuated this time‐dependent rightward shift and maintained the fluorescence distribution at markedly lower levels throughout the observation period, indicating that NP internalization is strongly dependent on dynamin‐mediated endocytic processes (Figure S6A). Because dynamin is involved in multiple endocytic pathways, including both clathrin‐ and caveolin‐mediated endocytosis, we further used chlorpromazine (CPZ), a widely used inhibitor of clathrin‐mediated endocytosis, to more directly assess the contribution of the clathrin pathway. CPZ treatment produced a pronounced leftward shift of the NPs‐associated fluorescence distribution at 2, 4, and 6 h compared with the corresponding control groups, indicating a marked reduction in NP internalization (Figure S6B). Together, these complementary pharmacological data indicate that NP uptake in MCF‐7 cells is strongly dependent on dynamin‐mediated endocytic mechanisms and further support a major contribution of clathrin‐mediated endocytosis to NP internalization.

Collectively, these findings establish that FAK ablation drives broad transcriptional reprogramming, resulting in coordinated changes to endocytosis‐associated gene networks. Analysis of the protein‐protein interaction network reinforces the role of FAK as a central integrator of signaling pathways, particularly those involving EGFR and RAC1. A key insight is the non‐canonical nature of this transcriptional control, as evidenced by the concurrent downregulation of the clathrin‐adaptor gene DAB2 and upregulation of other endocytic components (e.g., PACSIN2), coupled with the net increase in endocytic capacity, which indicates that FAK differentially regulates distinct endocytic pathways via a complex transcriptional network to maintain homeostasis. FAK loss disrupts this equilibrium, likely provoking a compensatory shift towards endocytic mechanisms more permissive for NP internalization.

### 2.7 FAK Depletion Enhances NP Accumulation via ECM Reduction

To evaluate the role of FAK in modulating tumor stroma and NP delivery in vivo, we established a FAK‐ knockout tumor model (Figure 7A). Histological analysis of tumor sections showed that FAK‐/‐ tumors exhibited markedly decreased expression of fibronectin and laminin, along with a reduction in collagen fiber density and organization as visualized by Masson's trichrome stain (Figure 7B). Western blot analysis of three independent tumor lysates confirmed substantially lower protein levels of fibronectin (FN1) and COL1A1 in FAK‐/‐ compared to WT tumors (Figure 7C). *Ex vivo* fluorescence imaging of tumors demonstrated that FAK‐/‐ tumors accumulated significantly stronger fluorescence of NPs than WT tumors, indicating FAK loss specifically promotes NP accumulation within tumors (Figure 7D,E). Assessment of NP penetration within decellularized tumor matrices via fluorescence imaging revealed a more profound and extensive signal distribution in FAK‐/‐ stroma, with intensity decay profiles showing a multi‐phasic, slower decline, consistent with enhanced matrix permeability (Figure 7F). Collectively, these in vivo findings establish that FAK ablation attenuates the tumor stromal barrier by suppressing the expression and impairing the architecture of key ECM proteins, which facilitates both the preferential accumulation and the deeper tissue penetration of systemically administered NPs.

**FIGURE 7:**
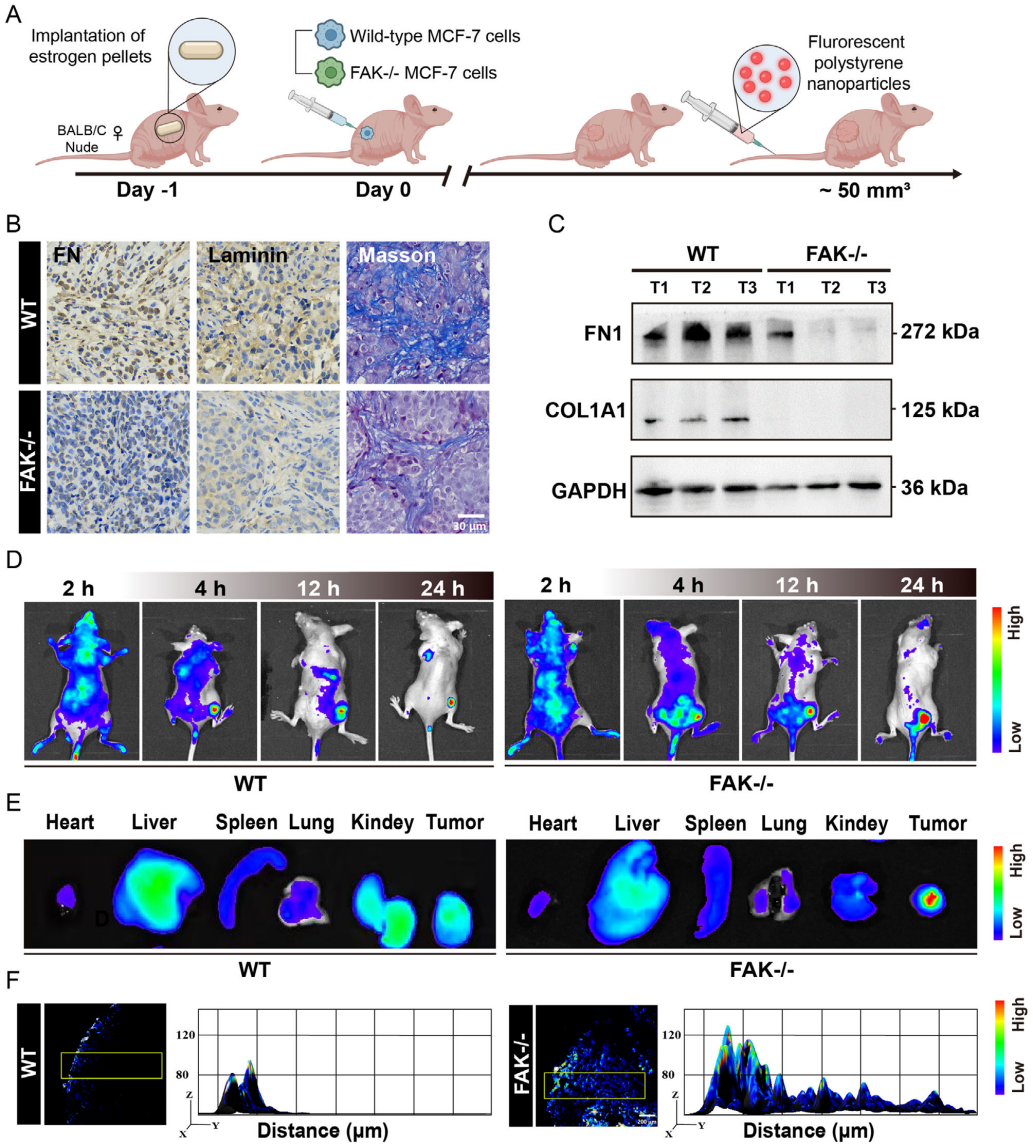
FAK deficiency remodels tumor ECM and enhances NP accumulation and penetration in vivo. (A) Schematic illustration of in vivo tumor model establishment and NP biodistribution analysis. (B) Tumor sections from WT and FAK‐/‐ groups were stained for fibronectin and laminin by immunohistochemistry, and with Masson's trichrome staining. Scale bar, 30 µm. (C) Western blot analysis of FN1 and COL1A1 protein levels in lysates from three independent WT and FAK‐/‐ tumor samples. GAPDH serves as a loading control. (D) In vivo biodistribution of NPs in tumor‐bearing mice. (E) Major organs including heart, liver, spleen, lung, kidney, and tumor tissues were collected for imaging. (F) Representative fluorescence images and reconstructed depth‐dependent fluorescence intensity profiles of nanoparticle penetration in decellularized WT and FAK‐/‐ tumor ECM.

### 2.8 In Vivo Antitumor Therapeutic Effect

Based on the central role of FAK in regulating both cellular and stromal mechanics, we constructed a FAK‐targeted nanotherapy system (DOX@PLGA/PEI‐PEG/siFAK). The NPs exhibited a uniform spherical morphology with an average hydrodynamic diameter of approximately 200 nm (Figure 8A; Figure S7). First, we assessed FAK protein and mRNA levels in transfected 4T1 cells to validate the gene‐silencing efficacy of siFAK, which demonstrated that specific siFAK sequences effectively reduced FAK expression (Figure 8B). In a tumor‐bearing mouse model, the animals were divided into four groups receiving treatments with saline, free DOX, siFAK‐loaded NPs, or combination DOX/siRNA NPs, respectively (Figure 8C). Tumors in the control (G1) groups progressed rapidly. Treatment with siFAK‐loaded NPs (G3) alone showed a modest but observable growth inhibition. Strikingly, the combination therapy group (G4) exhibited the most potent antitumor effect, with tumor growth being significantly suppressed compared to all other treatment groups (Figure 8D). This superior efficacy was visually confirmed at the experimental endpoint, where excised tumors from the G4 group were the smallest in size (Figure 8E). Immunohistochemical staining for key ECM components, COL1A1 and FN, revealed abundant expression in the control (G1) and free DOX (G2) groups. This expression was moderately reduced in the siFAK‐only group (G3) and was most markedly suppressed in the combination therapy group (G4), indicating that FAK silencing effectively attenuates ECM deposition and may alleviate the physical barrier within the tumor microenvironment. Consistent with these findings, hematoxylin and eosin (H&E) staining of tumor sections from the G4 group showed extensive necrosis, accompanied by disrupted tissue architecture, whereas the control groups exhibited densely packed and structurally intact tumor cells. These observations suggest that the combination treatment induces substantial tumor damage. To further assess cellular responses, Ki67 staining demonstrated a marked reduction in proliferative cells in the G4 group compared to all other groups, indicating effective suppression of tumor cell proliferation. In contrast, Tunel staining revealed a pronounced increase in apoptotic cells in the G4 tumors, confirming enhanced induction of programmed cell death (Figure 8F). These results demonstrate that combination therapy remodels the tumor ECM while concurrently suppressing proliferation and promoting apoptosis, validating the central hypothesis that disrupting FAK signaling to modulate the tumor stroma enhances conventional nano‐chemotherapy.

**FIGURE 8:**
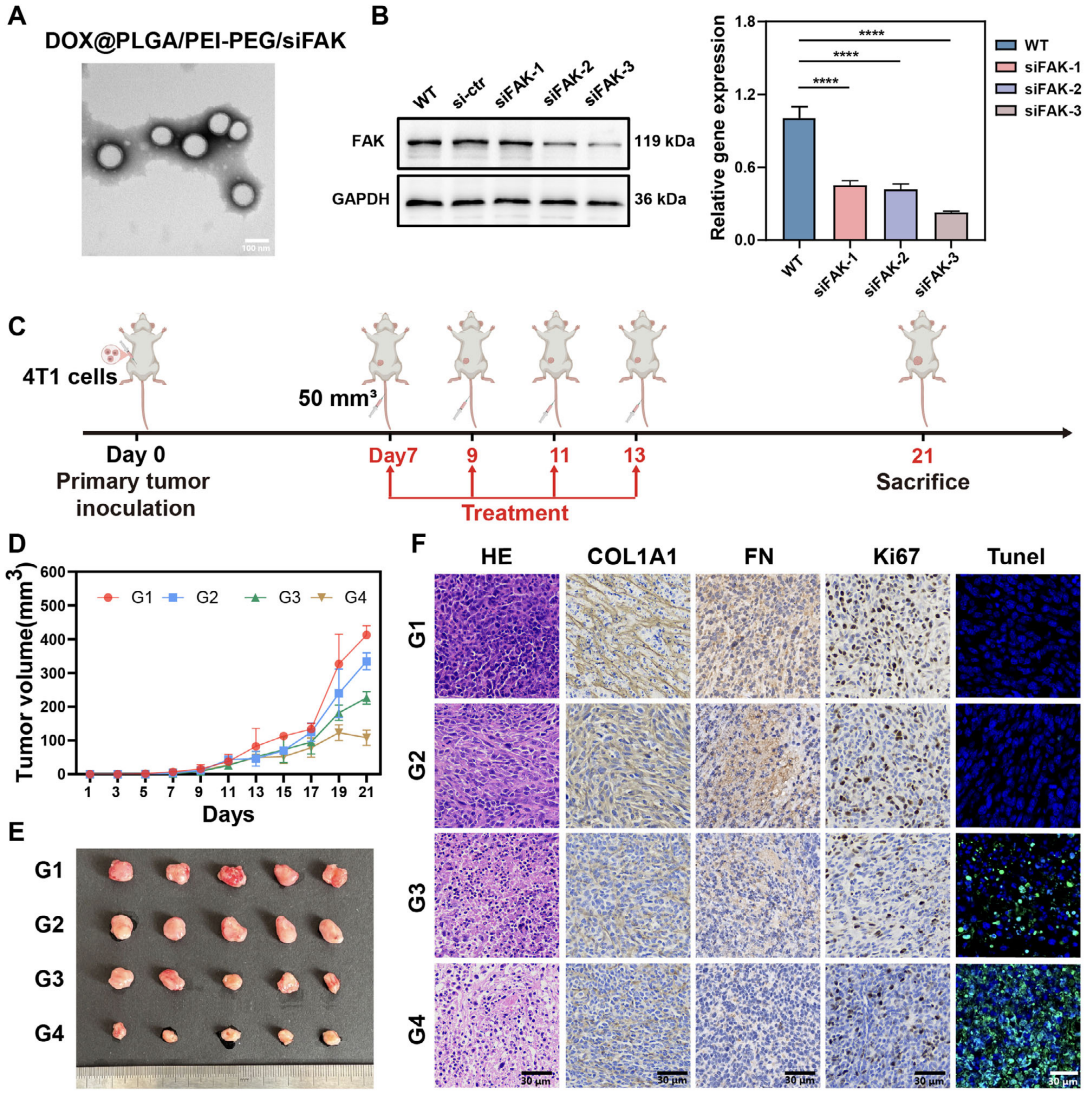
In vivo therapeutic evaluation of doxorubicin (DOX) and siFAK combinational nanotherapy in a 4T1 breast tumor model. (A) TEM image of the DOX@PLGA/PEI‐PEG/siFAK nanocomplex. Scale bar, 100 nm. (B) Analysis of FAK knockdown efficiency. Western blot analysis of FAK protein levels in 4T1 cells transfected with scrambled siRNA (si‐ctr) or three independent siFAK sequences. GAPDH served as a loading control (Left). qPCR analysis of FAK mRNA levels in 4T1 cells under the same transfection conditions (Right). Gene expression was normalized to GAPDH (*n* = 3 independent experiments). Statistical significance was calculated using one‐way ANOVA with Tukey's post hoc test, ^****^ *p* < 0.0001. (C) Experimental timeline of systemic treatments in orthotopic 4T1 tumor‐bearing mice. Mice were randomly assigned into four groups: saline control (G1), free DOX (G2), siFAK nanocomplex (G3), and DOX/siFAK combination nanocomplex (G4). (D) Tumor volume at the indicated time; mean ± SD. (E) Representative images of excised tumors at the experimental endpoint. Tumors were collected from each group at day 21 and photographed to compare gross morphology and size across treatment conditions. (F) Histopathological and immunohistochemical analysis of tumor tissues. Representative images of tumor sections stained for COL1A1, FN, H&E, the proliferation marker Ki67, and Tunel. Scale bars, 30 µm.

## 3 Discussion

This study reveals the multifaceted roles of FAK as a master regulator of the breast TME, highlighting its orchestration of a biophysical and signaling network that regulates nanotherapeutic delivery and efficacy. Our investigation began with the characterization of the primary physical barrier: utilizing decellularized matrices from patient‐derived samples, we directly observed that the dense and stiff ECM of breast tumors severely restricts the penetration of model NPs, corroborating the established concept that ECM biomechanics dictate therapeutic agent diffusion [1, 35, 36, 37, 38]. Beyond this structural impediment, we identify FAK as a critical upstream signaling hub whose activity is prognostically significant and centrally governs the restrictive properties of TME. Mechanistically, FAK exerts multi‐level control: it regulates ECM composition and organization (influencing collagen and fibronectin deposition), modulates cellular mechanical states (maintaining cytoskeletal integrity and stiffness), and tonically suppresses cellular endocytic capacity. Pharmacological or genetic targeting of FAK effectively remodels this tripartite barrier, simultaneously enhancing NP interstitial transport and cellular internalization. Based on this mechanistic insight, we rationally designed a combinatorial PLGA‐based nanoparticle co‐delivering siFAK and the chemotherapeutic doxorubicin (DOX). This therapeutic strategy synergistically dismantles the FAK‐maintained delivery barrier and attacks tumor cells, resulting in potent antitumor efficacy characterized by reduced ECM deposition, suppressed proliferation, and induced apoptosis. Our work thus reveals FAK inhibition as a potent strategy to overcome the pervasive challenge of drug penetration in solid tumors, translating a mechanistic understanding of stroma‐tumor signaling into a promising combinatorial nanomedicine.

FAK, a key mediator of ECM signaling, has emerged as a critical regulator of tumor progression by modulating cell adhesion and ECM remodeling [39, 40]. Our findings underscore the central role of FAK in regulating ECM homeostasis and tumor permeability. FAK deletion in MCF‐7 breast cancer cells led to significant changes in ECM composition, including decreased deposition of fibronectin and collagen. Notably, in monoculture, FAK depletion significantly reduced COL1A1 but did not significantly affect FN, owing to the limited capacity of MCF‐7 cells to independently assemble a robust FN matrix, whereas CAFs serve as the predominant source of COL1A1 and a major contributor to FN deposition. In the MCF‐7/CAF co‐culture model, FAK depletion resulted in marked reductions in both FN and COL1A1, likely reflecting altered tumor‐CAF paracrine communication and consequently decreased CAF‐associated ECM deposition. This reduction in ECM stiffness was accompanied by enhanced NP penetration in FAK‐deficient tumors, suggesting that FAK‐mediated regulation of ECM deposition plays a pivotal role in controlling ECM permeability. These results are consistent with previous reports that demonstrate the contribution of FAK signaling to ECM remodeling in cancer [8, 41].

Our data reveal that FAK governs NP delivery through two distinct yet complementary mechanisms: modulating the interstitial transport barrier and regulating cellular uptake capacity. This concept is consistent with recent evidence that mechanotransduction can directly regulate cell‐nanoparticle interactions. For example, inhibition of the mechanosensitive transcriptional regulator YAP was shown to enhance nanoparticle internalization in breast cancer cells through alterations in cell mechanics, membrane organization, and endocytic pathways [19]. In 3D models, a higher concentration of collagen inhibits the physical penetration of NPs, which aligns with the prevailing understanding in the field [1, 11, 37]. Notably, FAK inhibition enhanced both NP penetration and cellular internalization. This dual effect is mechanistically explained by our multiscale analysis. First, FAK ablation attenuated the stromal barrier in vivo, reducing the deposition of fibronectin, laminin, and collagen, which facilitated deeper NP penetration into tumors. Second, at the cellular level, FAK knockout induced a profound mechanical softness, disrupting actin cytoskeleton organization, reducing traction forces, and increasing membrane roughness. This mechanical reprogramming was coupled with broad transcriptional upregulation of genes across macropinocytosis, caveolin‐mediated, and clathrin‐mediated endocytic pathways. Crucially, functional validation confirmed clathrin‐mediated endocytosis as a dominant uptake route. We note that the enhancement of NP uptake following FAK depletion or inhibition was moderate in magnitude, consistent with FAK acting as a mechanoregulatory node rather than an exclusive determinant of endocytosis. This moderate effect is nonetheless biologically meaningful, as it reflects the relief of a mechanical constraint on cellular internalization and, when combined with improved ECM penetration, contributes to overall improvement in NP delivery. Thus, FAK constitutively restricts NP uptake by maintaining a stiff cellular phenotype and tonically suppressing endocytic capacity, whereas its inhibition dismantles both the extracellular and cellular barriers to nanotherapy. These results align with recent studies showing that FAK inhibitors can sensitize tumors to chemotherapy by enhancing the accessibility of therapeutic agents [42, 43].

RNA sequencing of FAK‐deficient MCF‐7 cells revealed a complex transcriptional reprogramming, with changes in genes associated with cytoskeletal organization, cell‐matrix adhesion, and endocytic trafficking. Notably, the upregulation of genes involved in actin dynamics (MYO15B, TPM2) and the downregulation of key endocytic genes (DAB2) suggest that FAK regulates not only ECM remodeling but also cellular processes crucial for drug uptake. The altered expression of these genes could provide insight into the mechanistic pathways by which FAK influences cellular morphology, adhesion, and NP internalization. Given that FAK is a key regulator of actin cytoskeletal dynamics, its depletion likely disrupts cellular architecture, which in turn enhances NP uptake by altering endocytic machinery. Our findings reveal that FAK functions as a key mechanistic node linking the physical properties of the tumor stroma to the capacity of tumor cells to internalize nanomedicines. By coordinating ECM remodeling, cellular mechanics, and endocytic activity, FAK regulates both the extracellular transport and intracellular uptake of NPs, thereby establishing a multiscale barrier to effective nanomedicine delivery. These findings extend the current understanding of how stromal architecture and cellular mechanical states jointly regulate nanomedicine trafficking in solid tumors and provide a mechanistic basis for targeting FAK to improve nanotherapeutic delivery.

Furthermore, our study highlights the importance of ECM remodeling in cancer therapy. We fabricated a PLGA‐based nanoparticle for co‐delivering siFAK and DOX. In vivo, this approach potently inhibited tumor growth by synergistically remodeling the tumor ECM (reducing collagen/fibronectin) and inducing direct cytotoxicity. While much of the research has focused on tumor cell‐targeted therapies, our findings support an emerging paradigm in mechanomedicine, in which pathological tissue mechanics are considered not merely as consequences of tumor progression but as actionable determinants that can be modulated to improve treatment efficacy [44, 45]. The mechanical and structural properties of the ECM, regulated by FAK, must be considered when designing nanomedicines and other cancer therapies. A better understanding of ECM mechanics and FAK signaling will be crucial for developing strategies that can overcome ECM‐mediated drug delivery barriers.

Beyond cancer nanotherapy, our findings may have implications for fibrotic diseases in which aberrant ECM deposition and mechanical feedback sustain disease progression. FAK is positioned at the interface between cell‐matrix adhesion and mechanotransduction, and recent studies have implicated FAK‐dependent signaling in the cellular response to pathological matrix mechanics [46]. In particular, fibroblast‐ECM interactions can establish a positive feedback loop in which matrix stiffening promotes fibroblast activation and further ECM deposition. Our finding that FAK inhibition reduces collagen and fibronectin accumulation therefore suggests that FAK may represent a mechanochemical target for disrupting this pathological cycle. Although the present study was performed in a tumor context, this framework raises the possibility that FAK‐directed strategies could be explored for ECM normalization and therapeutic delivery in fibrotic tissues. Our findings may also be relevant to immunotherapy, because the mechanical properties of the tumor ECM influence not only therapeutic drug transport but also immune‐cell access and function. Increasing evidence indicates that collagen‐rich and stiff tumor matrices can impede CD8^+^ T‐cell and NK‐cell infiltration and contribute to immune exclusion and dysfunction [47, 48]. Moreover, recent work in breast cancer has shown that a fibrotic microenvironment can induce collagen‐producing programs in tumor‐associated macrophages, further compromising CD8^+^ T‐cell function [47]. Thus, the FAK‐dependent reduction in ECM deposition observed here may create a more permissive physical environment for both nanomedicine penetration and immune‐cell trafficking. This provides a rationale for investigating FAK‐directed ECM remodeling in combination with immune checkpoint blockade or immunomodulatory nanoparticle delivery, although such immunotherapeutic effects remain to be experimentally established.

More broadly, our findings extend the concept of mechanotransduction from regulation of gene expression and cytoskeletal organization to the control of cellular material trafficking. FAK integrates extracellular mechanical cues through focal adhesions and coordinates downstream cytoskeletal and transcriptional responses. Recent work has shown that FAK, together with vinculin and talin, regulates mechanosensitive YAP localization, while recent studies in breast cancer further demonstrate that ECM mechanics can establish a YAP‐centered mechanotransduction loop that couples collagen organization and mechanical tension to collective tissue invasion [49, 50]. Together with our observation that FAK depletion alters cellular mechanics and endocytic programs, these findings suggest that FAK may function as a broader mechanochemical interface linking extracellular matrix mechanics to membrane trafficking. This concept may be relevant to developmental and pathological processes in which matrix mechanics, cell adhesion, cytoskeletal tension, and cellular trafficking must be coordinated to control tissue organization.

While our findings are compelling, there are several limitations to consider. First, our in vitro models, although useful for mechanistic insights, do not fully replicate the complexity of the TME in vivo. Further studies using more physiologically complex 3D systems and independent animal models will be necessary to validate our observations in a clinically relevant context. Second, while we focused on FAK as a key regulator, other signaling pathways may also contribute to the regulation of ECM and NP delivery. Future research should explore the crosstalk between FAK and other mechanotransduction pathways, such as those mediated by integrins and Rho GTPases, to provide a more comprehensive understanding of ECM remodeling in cancer. In addition, future studies should determine whether FAK‐dependent remodeling of the mechanical microenvironment can enhance immune‐cell infiltration or immunotherapy responses, and whether similar mechanisms operate in non‐malignant fibrotic tissues. It will also be important to distinguish tumor‐cell‐intrinsic FAK functions from stromal FAK signaling and to establish whether selective or spatially restricted FAK inhibition can preserve physiological mechanotransduction while effectively disrupting pathological matrix remodeling.

## 4 Conclusions

This study establishes that the stiff, dense tumor ECM is a major physical barrier to nanomedicine delivery and reveals FAK as a master regulator of ECM remodeling and cellular mechanics in breast cancer models (Figure 9). FAK loss or inhibition synergistically enhances delivery by increasing tissue permeability and upregulating endocytic uptake. An FAK‐targeted combinatorial nanoplatform co‐delivering siRNA and doxorubicin confirmed potent antitumor efficacy in vivo. Collectively, these findings identify FAK as a key mechanotransductive node and a promising therapeutic target for dismantling both stromal and cellular mechanical barriers that limit anticancer efficacy.

**FIGURE 9:**
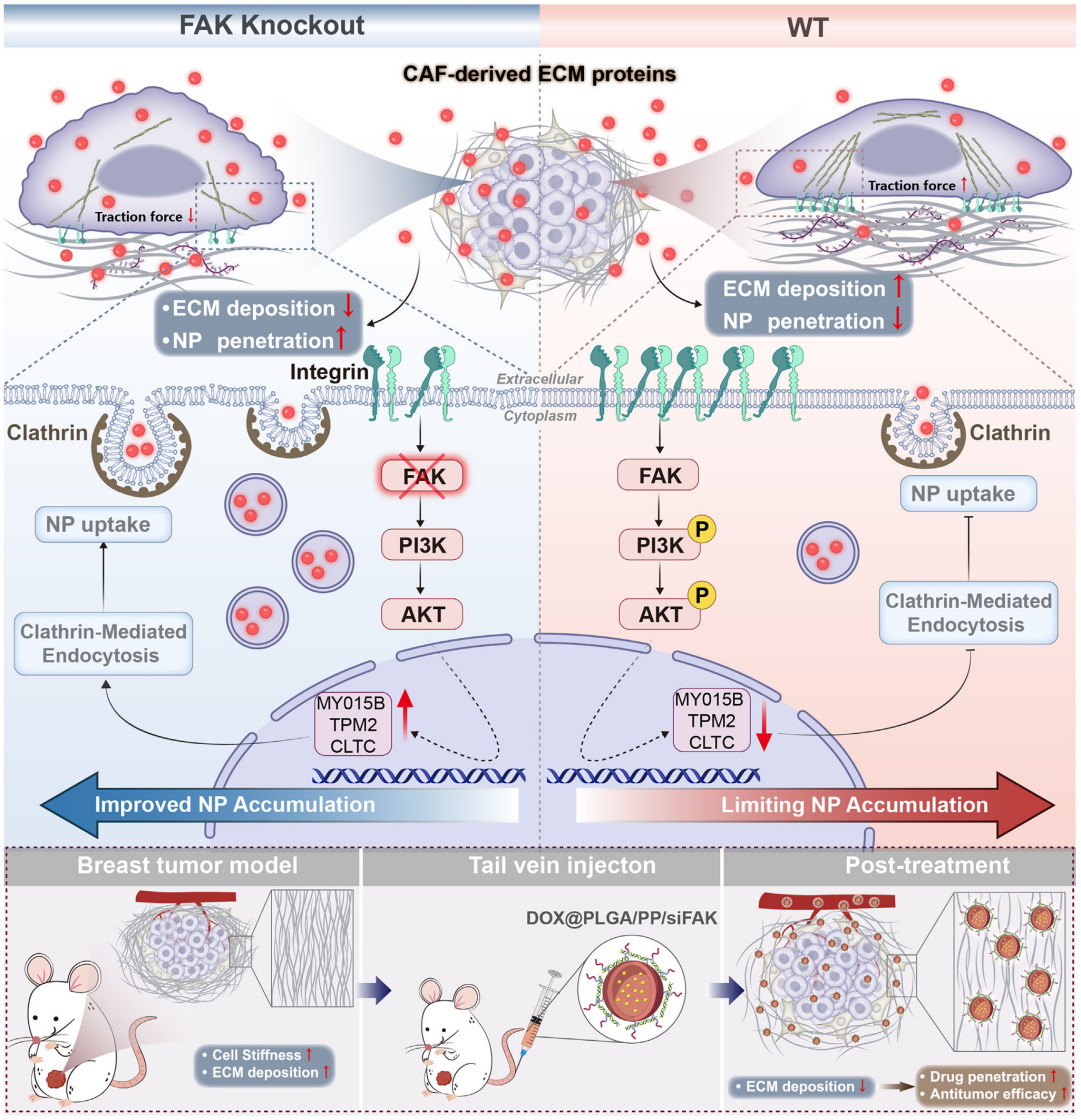
Schematic representation of FAK‐mediated ECM remodeling and NP uptake. Left, FAK knockout cells exhibit a rounder morphology with abundant membrane protrusions and microvilli, reduced cell spreading, and lower cellular traction forces, indicative of lower cell stiffness and a mechanically compliant phenotype. Loss of FAK suppresses the FAK/PI3K/AKT signaling pathway, resulting in reduced ECM deposition despite co‐culture with CAFs. Meanwhile, FAK deficiency alters endocytosis‐related genes, enhances clathrin‐mediated endocytosis, and promotes intracellular NP uptake. The reduced collagen‐rich ECM further facilitates NP penetration, ultimately leading to increased NP accumulation within tumors. Right, WT cells display a well‐spread morphology with enhanced cytoskeletal organization and higher cellular traction forces, corresponding to a stiffer cellular and extracellular mechanical state. Active integrin‐mediated FAK signaling activates the PI3K/AKT pathway, promotes CAF‐mediated ECM deposition and collagen remodeling, and generates a dense ECM network that acts as a physical barrier to NP penetration. Concurrently, FAK signaling suppresses endocytosis‐related gene expression and limits clathrin‐mediated NP uptake. In vivo, systemic administration of DOX@PLGA/PEI‐PEG/siFAK remodels the tumor microenvironment by reducing ECM deposition, thereby improving intratumoral drug penetration and enhancing antitumor efficacy. Collectively, FAK coordinates ECM remodeling and cellular endocytosis to establish a mechanical barrier that restricts nanoparticle accumulation in solid tumors.

## 5 Materials and Methods

### 5.1 Cell Lines and Cancer‐associated Fibroblasts (CAFs) Isolation

Human MCF‐7 cells (DMEM, 10% FBS, 1% P/S; 37°C, 5% CO_2_) were used. Cells were provided by HySigen Biosciences Inc. CAFs were isolated from fresh breast invasive ductal carcinoma specimens (The Affiliated Cancer Hospital of UESTC; ethical approval obtained). Tissue was minced, digested with collagenase I (100 U/mL) and hyaluronidase (150 U/mL) for 6–8 h at 37°C, filtered, and enriched by differential trypsinization. CAFs were co‑cultured with MCF‐7 in direct‑contact systems.

### 5.2 Tissue Decellularization

Normal and tumor tissue samples from 5 patients resected during clinical surgery were placed in PBS solution at 4°C and transferred to the laboratory within 4 h. After brief washing with phosphate‐buffered saline (PBS), the biopsy tissues were cut into 3.0 × 3.0 × 3.0 mm^3^ pieces and transferred to 1% SDS solution and gently shaken at room temperature for 48 h. The tissues were thoroughly rinsed with DPBS and gently shaken for a total of 16 h, with the buffer replaced after 8 h (two washes in total). Finally, the ECM was preserved in PBS solution containing 100 U/mL penicillin/streptomycin antibiotics at 4°C for subsequent analysis.

### 5.3 Histology and Immunofluorescence

After deparaffinization and rehydration, formalin‐fixed, paraffin‐embedded (FFPE) sections were treated with antigen retrieval buffer and subjected to Masson's trichrome staining (Servicebio G1006) or immunohistochemistry (IHC). For IHC, sections were incubated with primary antibodies against FAK (CST 71433T), FN (ET1702‐25), laminin (ab11575), COL1A1 (HA722517), or Ki67 (abcam ab16667) for 1 h, followed by incubation with an HRP‐labeled secondary antibody for 1 h, DAB development, and hematoxylin counterstaining. For the TUNEL (G1504‐100T) assay, sections were incubated with the TUNEL reaction mixture, counterstained with DAPI, and visualized by fluorescence microscopy. For cell immunofluorescence, MCF‐7 cells were fixed (4% PFA, 15 min), permeabilized (0.4% Triton X‑100, 5 min), blocked (1% BSA), and incubated with primary antibodies (anti‑COL1A1, anti‑FN; overnight, 4°C), followed by Alexa‑fluorophore secondaries, phalloidin‑555 (F‑actin), and DAPI. Confocal imaging was performed using a Zeiss LSM980.

### 5.4 Mechanical Measurements

Tissue stiffness: 20‑µm cryosections were measured by AFM (Bruker MultiMode 8, force‑volume mode, Si_3_N_4_ probe, k = 0.1 N/m, tip radius 20 nm, loading 1 µm/s, max force 200 nN). Young's modulus was extracted via the Hertz model (conical indenter, ν = 0.5). Cellular traction: PAAm gels (Young's modulus 40 kPa) with 0.5 µm fluorescent beads were coated with fibronectin (50 µg/mL). Single cells (500 cells/cm^2^, 48 h adhesion) were imaged before and after lysis (5% SDS). Displacements were computed by PIV and FTTC (regularization 2 × 10^−^^9^, ν = 0.5).

### 5.5 NP Penetration/Diffusion/Internalization Assays

Microcontact printing and tumor spheroid formation. For microcontact printing, a micropatterned polydimethylsiloxane (PDMS) stamp containing circular micropatterns with a diameter of 100 µm was ultrasonically cleaned and dried at 37°C. The 40 µg/mL fibronectin mixture (200 µL) was applied onto the stamp and incubated for 1 h. After removal of excess liquid, the stamp was gently pressed onto a pre‐prepared culture dish to transfer fibronectin onto the substrate. The culture dish was subsequently treated with 1% (w/v) F127 for 1 h, washed three times with ultrapure water, and UV‐sterilized for 1 h. Cells were then seeded onto the micropatterned substrate to form tumor spheroids.

NP penetration and diffusion assays. (i) Microfluidic channels: collagen I (0.75, 1.0, 1.5 mg/mL) polymerized at 37°C; Cy5‑PS NPs (50 µg/mL) diffused from one reservoir for 2 h; fluorescence intensity profiles were normalized. (ii) Decellularized ECM scaffolds: tissues incubated with NPs (48 h), fixed, cryoprotected (10%–30% sucrose), OCT‑embedded, sectioned (10 µm). (iii) Spheroid uptake: MCF‐7 spheroids formed in low‑adhesion V‑bottom plates (4 d) were exposed to NPs (50 µg/mL, 2 h), dissociated, and analyzed by flow cytometry. (iv) Collagen‑embedded spheroids: spheroids were cast in 0.5 or 2 mg/mL collagen I (72 h), then NPs were added; penetration was assessed by fluorescence microscopy.

NP internalization. WT and FAK‐/‐ MCF‐7 cells were co‑seeded (1 × 10^5^ each) in 24‑well plates, incubated with NPs (50 µg/mL, 4 h), and analyzed by flow cytometry (Agilent) and fluorescence microscopy (Nikon Eclipse Ti2).

### 5.6 Molecular Pathway Analyses

RNA isolation and quantitative PCR. Total RNA was extracted using the FastPure Cell/Tissue Total RNA Kit (Vazyme, Nanjing, China, RC112‐01) according to the manufacturer's protocol. cDNA was synthesized from total RNA using a reverse transcription kit (Vazyme, Nanjing, China, R323). Quantitative PCR (*q*PCR) was performed with the cDNA and specific primers using SYBR Green Master Mix (Vazyme, Nanjing, China, Q712). The primers used are listed in Table S1.

RNA sequencing. RNA‐seq libraries were prepared by Berry Genomics Company (Beijing), and paired‐end sequencing was performed on Illumina NovaSeq 6000. Differentially expressed genes were identified with *p* < 0.01. Pathway enrichment analysis was carried out using GSEA, http://software.broadinstitute.org/gsea/index.jsp.

Transcriptomic statistical analysis: transcriptomic data from FAK‐/‐ and WT samples were analyzed on a gene‐by‐gene basis using two‐sided unpaired Student's *t*‐tests. Log2 fold changes (log2FC) were calculated from the mean expression levels between the two groups. Raw *P*‐values were adjusted using the Benjamini‑Hochberg (BH) method to control the false discovery rate (FDR). Genes with |log2FC| ≥ 1 and FDR‐adjusted *p* < 0.05 were considered differentially expressed. For heatmap visualization, genes were ranked according to the combined magnitude of log2FC and statistical significance, and the top 50 genes were selected.

### 5.7 Scanning Electron Microscopy (SEM)

WT and FAK‐/‐ MCF‐7 cells on coverslips were fixed (3% glutaraldehyde), dehydrated (30%‐100% ethanol), Pd‑coated, and imaged at 5 kV (FE‐SEM, Thermo Scientific Apreo 2 S, Thermo Fisher Scientific, USA).

### 5.8 Statistics

Data are presented as mean ± SD. For comparisons between two groups, an unpaired *t*‐test was employed. For comparisons among multiple groups, one‐way ANOVA followed by Tukey's post hoc test. *P <* 0.05 was considered statistically significant.

### 5.9 Ethics Statement

The collection and use of human tissue samples in this study were approved by the Ethics Committee of the University of Electronic Science and Technology of China (UESTC) (approval number: 106142023121327795). Clinical samples were collected and used only with patient consent. All animal experiments were conducted with approval from the Ethics Committee of UESTC (approval number: 10642024091930925), in strict accordance with the ARRIVE guidelines. Mice were housed in specific pathogen‐free conditions with ad libitum access to food and water.

## Author Contributions

**Tian Zhao**: conceptualization, writing – original draft, methodology, validation, visualization, writing – review and editing, formal analysis, software. **Hao Wu**: visualization, writing – original draft, conceptualization. **Yujie Qin**: methodology, validation, software. **Jingfei Zhu**: methodology, validation. **Ye Huang**: visualization, software, writing – original draft. **Wei Chen**: software. **Jiaxuan Yu**: formal analysis, software. **Xiang Qin**: supervision. **Shun Li**: supervision. **Junjie Li**: supervision, resources. **Tingting Li**: data curation, supervision, funding acquisition, conceptualization, writing – review and editing, investigation. **Yiyao Liu**: funding acquisition, project administration, resources, supervision, conceptualization, investigation, writing – review and editing.

## Conflicts of Interest

The authors declare no conflicts of interest.

## Supporting information




**Supporting File**: advs78146‐sup‐0001‐SuppMat.docx.

## Data Availability

The data that support the findings of this study are available from the corresponding author upon reasonable request.
