# Supplementary material for: FAK Mediated Coordination of Extracellular Matrix Remodeling and Cellular Mechanics Impairs Nanomedicine Delivery in Solid Tumors
Source: Adv Sci (Weinh). 2026 Oct 14:e78146. Online ahead of print. doi: 10.1002/advs.78146 (PMC13668767; doi:10.1002/advs.78146)
Supplement: Supplementary file 1 [file ADVS-9999-e78146-s001.docx]

**FAK Mediated Coordination of Extracellular Matrix Remodeling and Cellular Mechanics Impairs Nanomedicine Delivery in Solid Tumors**

Tian Zhao ^1^, Hao Wu ^2^, Yujie Qin ^1^, Jingfei Zhu ^1^, Ye Huang ^1^, Wei Chen ^1,2^, Jiaxuan Yu ^1^, Xiang Qin ^1^, Shun Li ^1^, Junjie Li ^1*^, Tingting Li ^1 *^, Yiyao Liu ^1,2,3,4,5 *^

*^1^ Department of Breast Surgeryt, Sichuan Clinical Research Center for Cancer, Sichuan Cancer Hospital & Institute, Sichuan Cancer Center, and School of Life Science and Technology, University of Electronic Science and Technology of China, Chengdu, Sichuan, People’s Republic of China*

*^2^ TCM Prevention and Treatment of Metabolic and Chronic Diseases Key Laboratory of Sichuan Province, Hospital of Chengdu University of Traditional Chinese Medicine, Chengdu, Sichuan, People’s Republic of China*

*^3^ Department of Otorhinolaryngology, Hospital of Chengdu University of Traditional Chinese Medicine, Chengdu, Sichuan, People’s Republic of China*

*^4^ Department of Urology, Deyang People's Hospital, Deyang, Sichuan, People’s Republic of China*

*^5^ The Department of Head and Neck Surgery, Cancer Hospital of Shantou University Medical College, Shantou, Guangdong, People’s Republic of China*

***Corresponding authors**:

Email addresses: [lijunjie@scszlyy.org.cn](mailto:lijunjie@scszlyy.org.cn)(J. Li), [litingting@uestc.edu.cn](mailto:litingting@uestc.edu.cn) (T. Li); [liuyiyao@uestc.edu.cn](mailto:liuyiyao@uestc.edu.cn) (Y. Liu)

Supporting Information

Supplementary Note 1. Figures

**Fig S1. FAK modulates ECM protein expression**. (**A**) Representative immunofluorescence images of WT and FAK-/- MCF-7 cells. Cells were stained for COL1A1 (red) and counterstained with DAPI (blue) for nuclei. Scale bars, 10 μm. (**B**) Quantitative statistical analysis corresponding to (A). Data are presented as mean ± SD, *n* = 3 independent experiments; *****P* < 0.0001, unpaired *t*-test. (**C**) Representative immunofluorescence images of WT and FAK-/- MCF-7 cells. Cells were stained for FN (red) and counterstained with DAPI (blue) for nuclei. Scale bars, 10 μm. (**D**) Quantitative statistical analysis corresponding to (C). Data are presented as mean ± SD, *n* = 3 independent experiments; ns indicates no significant difference, unpaired *t*-test.

**Fig S2. FAK modulates ECM protein expression**. (**A**) Representative immunofluorescence images showing COL1A1 (green) expression in WT or FAK-/- MCF-7 cells co-cultured with CAFs. F-actin was stained with phalloidin (red), and nuclei were counterstained with DAPI (blue). Scale bars, 20 μm. (**B**) Representative immunofluorescence images showing FN (green) expression in WT or FAK-/- MCF-7 cells co-cultured with CAFs. Nuclei are stained with DAPI (blue). Scale bars, 20 μm.

**Fig S3. Collagen Density Regulates Nanoparticle Penetration in 3D Tumor Spheroids**. (**A**) Schematic illustration of the experimental workflow for generating 3D tumor spheroids and embedding them in collagen matrices. (**B**) Representative reflectance imaging and corresponding fluorescence microscopy images showing tumor spheroids embedded in either low-density (0.5 mg/mL) or high-density (2.0 mg/mL) collagen matrices. Collagen fibers were visualized by reflectance microscopy. Scale bar, 50 μm. The red fluorescence signal indicates the distribution of NPs in 3D tumor spheroids. Scale bars, 100 μm.

**Fig S4. Analysis of PS NP diffusion in collagen matrices of varying densities**. (**A**) Schematic illustration showing the diffusion of NPs within a 3D collagen matrix. (**B**) Representative fluorescence microscopy images showing the spatial distribution of red fluorescent NPs within collagen hydrogels at indicated concentrations (0.75, 1.0, 1.5 mg/mL). Scale bar, 100 µm.

**Fig S5. FAK depletion alters cell morphology and actin cytoskeleton organization**. (**A**) Representative immunofluorescence images showing F-actin (phalloidin, red) and nuclei (DAPI, blue) in WT and FAK-/- MCF-7 cells. Merged images are shown on the right. Scale bars, 40 μm. (**B**) Quantification of cell area for WT and FAK-/- MCF-7 cells. Data are presented as mean ± SD (*n* = 3 independent experiments); *****P* < 0.0001, unpaired *t*-test. (**C**) Scanning electron microscopy images of WT and FAK-/- MCF-7 cells. Representative images of three individual cells are shown for each condition, along with a wide-field view of the cell morphology. Scale bars, 10 μm (left) and 50 μm (right).

**Fig S6. Clathrin-mediated endocytosis contributes to NP uptake in MCF-7 cells**.

(A) MCF-7 cells were incubated with fluorescent PS NPs in the presence or absence of Dynasore (80 μM), a dynamin inhibitor, and intracellular NPs-associated fluorescence was analyzed by flow cytometry at 2, 4, and 6 h post-incubation. (B) MCF-7 cells were incubated with fluorescent PS NPs in the presence or absence of chlorpromazine (CPZ; 10 μg/mL), a widely used inhibitor of clathrin-mediated endocytosis. The x-axis represents fluorescence intensity (arbitrary units, a.u.), and the y-axis represents cell count.

**Fig S7. Characterization of DOX@PLGA/PEI-PEG/siFAK nanosystem**. (**A**) Hydrodynamic diameter distributions of PLGA-DOX and DOX@PLGA/PEI-PEG nanoparticles measured by dynamic light scattering (DLS). Data are presented as mean ± SD (*n* = 3). (**B**) Gel electrophoresis (agarose gel) image showing the ability of NPs to form complexes with siRNA at different weight ratios (DOX@PLGA/PEI-PEG: siRNA). "Naked siRNA" represents the control without any treatment.

Supplementary Note 2. Materials and Methods

## ***1. Tumor Spheroid and 3D Model Construction***

Tumor Spheroid Generation: Generated in F127-treated low-adhesion V-bottom 96-well plates via centrifugation (1,500 rpm for 1 min) and cultured for 4 days. Collagen-Embedded Models: Spheroids were embedded in Type I collagen matrices (0.5 or 2 mg/mL) in custom cylindrical molds. Structural Characterization: Cell-free collagen matrices were imaged using a ZEISS LSM980 confocal microscope. Fibril alignment and coherence indices were quantified using the ImageJ plugin OrientationJ.

## ***2. Western blotting***

Cells were lysed with RIPA buffer (Merck Millipore, Germany) containing 1% protease and phosphatase inhibitor cocktails on ice, and then centrifuged at 14,000 g for 15 min at 4℃. The supernatants were collected, and protein concentrations were determined using the Bicinchoninic Acid (BCA protein assay kit, Solarbio, Beijing, China) protein assay, and 20 μg of protein per sample were loaded onto 10% polyacrylamide gels and run at 80 V. Proteins were transferred to a polyvinylidene difluoride membrane using the Trans-Blot Turbo transfer system (Bio-Rad, USA). The membranes were blocked with 5% BSA in TBST and incubated with primary antibodies diluted in 5% BSA in TBST overnight at 4℃. They were then probed with proper HRP-linked secondary antibodies for 2 h at room temperature. Chemiluminescence was detected using the ChemiDoc imaging system (Bio-Rad, USA), and band intensities were quantified using Bio-Rad Image Lab software. The primary antibodies used were rabbit anti-FAK (4912, Cell Signaling Technology), rabbit anti-pPI3K (4228t, Cell Signaling Technology), rabbit anti-pAKT (62471, GenuIN Biotech), rabbit anti-COL1A1 (HA722517, HUABIO), rabbit anti-Fibronectin (ET1702-25, HUABIO).

## ***3. In vivo tumor permeability and nanoparticle biodistribution study***

Female BALB/c nude mice (6–8 weeks old) were used to establish subcutaneous xenograft models. To support the growth of estrogen-dependent MCF-7 cells, mice were implanted subcutaneously with estradiol pellets one day prior to tumor inoculation. Subsequently, 5 × 10^6^ WT or FAK-/- MCF-7 cells suspended in PBS were injected subcutaneously into the dorsal flank of each mouse. Tumor growth was monitored every three days using a digital caliper, and tumor volume was calculated as V = (length × width²)/2. When tumors reached approximately 50 mm³, Cy5-labeled fluorescent PS NPs were administered via tail vein injection. At the designated endpoint, mice were euthanized and major organs (heart, liver, spleen, lung, and kidney) together with tumor tissues were harvested for *ex vivo* fluorescence imaging to evaluate nanoparticle biodistribution. Tumor sections and decellularized tumor ECM scaffolds were further analyzed by fluorescence microscopy to assess nanoparticle accumulation and penetration within the tumor microenvironment.

For therapeutic studies, orthotopic 4T1 breast tumor models were established by injecting 1 × 10⁶ 4T1 cells into the mammary fat pad of female BALB/c mice. Tumor growth was monitored until volumes reached approximately 50 mm³. Mice were then randomly assigned into four treatment groups: saline control (G1), free doxorubicin (G2), siFAK-loaded nanocomplex (G3), and DOX/siFAK co-loaded nanocomplex (G4). Systemic treatments were administered via tail vein injection on days 7, 9, 11, and 13 post randomization. Tumor volumes were measured throughout the study, and all animals were sacrificed on day 21 for downstream analyses, including tumor collection and ex vivo evaluation.

## 4. ***Preparation of DOX@PLGA/EI-PEG/siFAK Nanocomplexes***

PVA solution was prepared by dissolving PVA in preheated deionized water at 65 °C and stored at 4 °C after cooling. PEI–PEG (PP) was synthesized by EDC/NHS-mediated coupling: NH₂-PEG-COOH (36 mg) was activated with EDC (7.4 mg) and NHS (5.8 mg) for 3 h, followed by addition of PEI (32 mg) and overnight reaction at room temperature. The product was purified by dialysis using a 5 kDa MWCO membrane for 72 h and lyophilized to obtain PP. DOX@PLGA nanoparticles were prepared using a W/O/W double-emulsion solvent-evaporation method. Briefly, PLGA (100 mg) was dissolved in 2 mL DCM and emulsified with DOX·HCl (6 mg in 0.2 mL water), followed by sonication and emulsification in 10 mL PVA solution. After solvent evaporation for 12 h, nanoparticles were collected by centrifugation and washing. PP was subsequently conjugated onto the nanoparticle surface at a PLGA/PP weight ratio of 24:1 using EDC/NHS. Finally, siFAK was electrostatically adsorbed onto PLGA/PP nanoparticles at a PLGA/PP:siFAK weight ratio of 200:1 for 30 min at room temperature, yielding DOX@PLGA/PP/siFAK nanocomplexes.

## 5. ***siRNA Loading Capacity***

The siRNA loading capacity of the NPs was evaluated using agarose gel retardation assay. Briefly, DOX@PLGA/PEI-PEG NPs were incubated with siRNA at various mass ratios (100:1, 150:1, 200:1, and 300:1, NPs:siRNA, w/w) in RNase-free water at room temperature for 30 min to allow complexation. The resulting complexes (equivalent to 0.5 µg of siRNA per lane) were mixed with 6× loading dye and electrophoresed on a 2% (w/v) agarose gel containing 0.5 µg mL⁻¹ ethidium bromide in 1× TBE buffer (diluted from TBE (5×), Beyotime, Cat# ST718) buffer at 120 V for 20 min. Naked siRNA was used as a free control. After electrophoresis, the gel was visualized under a UV transilluminator (Gel Doc XR+, Bio-Rad). Successful binding of siRNA to the NPs was indicated by the retention of siRNA in the loading wells, whereas uncomplexed siRNA migrated freely into the gel.

**Table S1. List of primers used in this study**

| **Gene name** | **Primer sequence** |
| --- | --- |
| *GAPDH* (human) | F-ACAACTTTGGTATCGTGGAAGG |
|  | R-GCCATCACGCCACAGTTTC |
| *LAMININ* (human) | F-GTCACAGAGCAGGAGGTGGCT |
|  | R-GCTTCTGTCAAGACTCTCCAGG |
| *FN1* (human) | F-ACAACACCGAGGTGACTGAGAC |
|  | R-GGACACAACGATGCTTCCTGAG |
| *COL1A1* (human) | F-GATTCCCTGGACCTAAAGGTGC |
|  | R-AGCCTCTCCATCTTTGCCAGCA |
| *CLTC* (human)  *PTK2* (human)  *PTK2* siRNA#1 (mouse)  *PTK2* siRNA#2 (mouse)  *PTK2* siRNA#3 (mouse) | F-GTCTTTCTCGCTACCTGGTACG  R-GGTCCTGAGTCTCAGACAAAGC  F-TGAATTTCTTCTATCAACAG  R-CCTGGCTTCATCTATTCCAT  CCAACCTTAATAGAGAAGAAA  GCCTTAACAATGCGTCAGTTT  CCTGGCATCTTTGATATTATA |
